# Cytotoxic Tetrahydroxanthone Dimers from the Mangrove-Associated Fungus *Aspergillus versicolor* HDN1009

**DOI:** 10.3390/md16090335

**Published:** 2018-09-14

**Authors:** Guihong Yu, Guangwei Wu, Zichao Sun, Xiaomin Zhang, Qian Che, Qianqun Gu, Tianjiao Zhu, Dehai Li, Guojian Zhang

**Affiliations:** 1 Key Laboratory of Marine Drugs, Chinese Ministry of Education, School of Medicine and Pharmacy, Ocean University of China, Qingdao 266003, China; Yuguihong1990@126.com (G.Y.); gweiwu@163.com (G.W.); 171774170@163.com (Z.S.); xiaominzhang91@163.com (X.Z.); cheqian064@ouc.edu.cn (Q.C.); guqianq@ouc.edu.cn (Q.G.); zhutj@ouc.edu.cn (T.Z.); dehaili@ouc.edu.cn (D.L.); 2 Laboratory for Marine Drugs and Bioproducts of Qingdao Pilot National Laboratory for Marine Science and Technology, Qingdao 266237, China; 3 Open Studio for Druggability Research of Marine Natural Products, Qingdao Pilot National Laboratory for Marine Science and Technology, Qingdao 266237, China

**Keywords:** mangrove-derived fungus, *Aspergillus versicolor*, tetrahydroxanthone dimers, antibacterial activity, cytotoxicity

## Abstract

Three new tetrahydroxanthone dimers, 5-*epi*-asperdichrome (**1**), versixanthones N (**2**), and O (**3**), were isolated from the mangrove-derived fungus *Aspergillus versicolor* HDN1009. Their structures, including the absolute configurations, were elucidated by NMR, HRMS, and circular dichroism (CD) experiments. Among them, compound **1** was the second example of tetrahydroxanthone dimers, which dimerized by a rare diaryl ether linkage and showed promising antibacterial activities against *Vibrio parahemolyticus*, *Bacillus subtilis*, *Mycobacterium phlei*, and *Pseudomonas aeruginosa*, with MIC values ranging from 100 μM to 200 μM; whilst compounds **2** and **3** exhibited extensive cytotoxicities against five cancer cell lines (HL-60, K562, H1975, MGC803, and HO-8910), with IC_50_ values ranging from 1.7 μM to 16.1 μM.

## 1. Introduction

Xanthones are a family of 9*H*-xanthen-9-one derivatives, which are widely distributed in plants, fungi, and lichens. The structures typically occur as either fully aromatized or aliphatic cyclic ketones with dihydro-, tetrahydro- or hexahydro- modifications, and poly-substitutions [1]. Among those structures, tetrahydroxanthone dimers, which were usually dimerized through biaryl C-C single bond with diverse dimeric patterns, such as 2–2′, 2–4′, 4–2′, and 4–4′ linkages [2], were frequently reported for their intriguing structures and impressive activities (antimicrobial, antiplasmodial, and antitumor bioactivities) [2,3,4]. However, tetrahydroxanthone dimers linked by diaryl ether bond were relatively rare, with only one instance reported [5].

In our previous study, the mangrove-associated fungus *Aspergillus versicolor* HDN1009 has been proven to be a fruitful producer of various xanthone analogues [2,6]. In a recent work, UPLC-MS and cytotoxicity directed fractionation method were employed in a new trial of chemical study on HDN1009, to track unique xanthone structures. By carefully examining the UV absorption, predicted molecular weight, and the bioactivities of the unique peaks, three new tetrahydroxanthone dimers, named 5-*epi*-asperdichrome (**1**), versixanthones N (**2**), and O (**3**), were discovered and isolated, amongst which, 5-*epi*-asperdichrome (**1**) was the second example of tetrahydroxanthone dimers linked by a diaryl ether bond, which showed mild activities against four kinds of bacterial strains. Versixanthones N (**2**) and O (**3**) showed extensive cytotoxicities against five cancer cell lines. Herein, we will report the details of isolation, structure elucidation, and bioactivity evaluation of the above compounds. 

## 2. Results and Discussion

**Figure 1:**
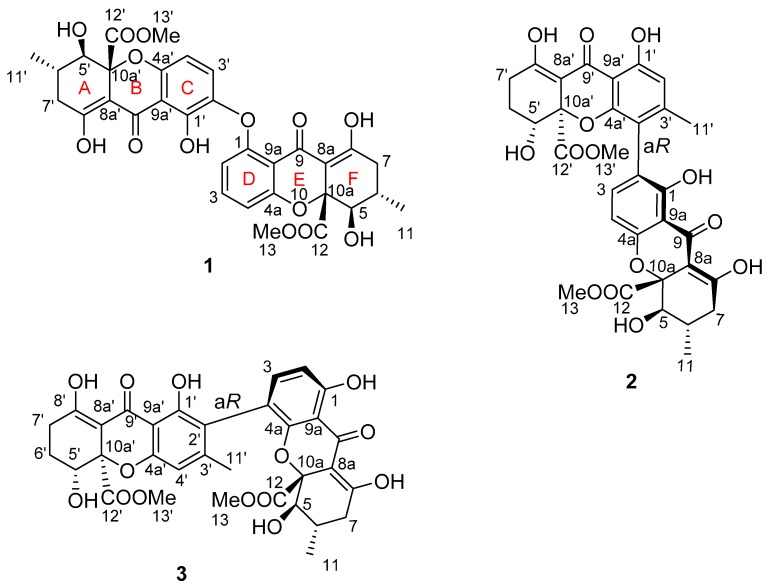
Structures of compounds **1**–**3**.

The *Aspergillus versicolor* HDN1009 was cultured (50 L) under static conditions at 28 °C for 4 weeks, as previous reported in Reference [2]. Guided by UPLC-MS data, the EtOAc extract (30 g) of the fermentation was fractionated by silica gel vacuum liquid chromatography, C-18 ODS column chromatography, Sephadex LH-20 column chromatography, ODS MPLC, and finally HPLC to yield compounds **1** (4.0 mg), **2** (3.0 mg), and **3** (4.0 mg) (Figure 1).

**Figure 2:**
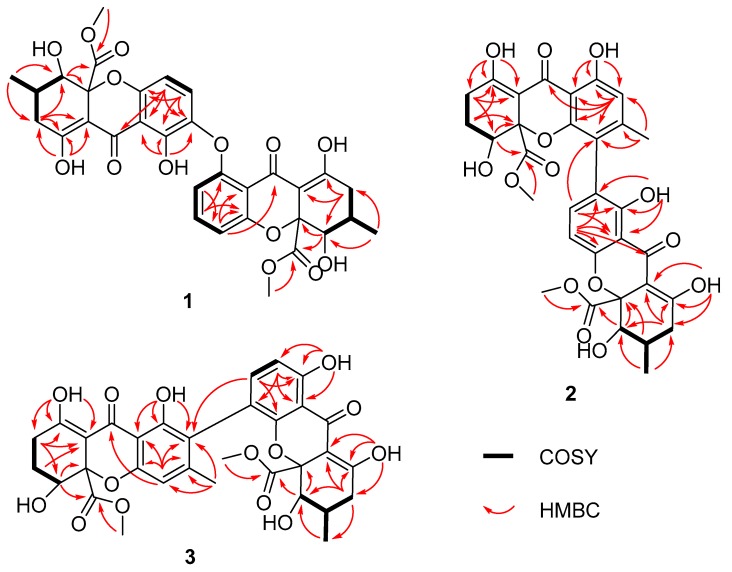
Key HMBC and ^1^H-^1^H COSY correlations of **1**–**3**.

Compound **1** was isolated as a pale, yellow powder, with a molecular formula of C_32_H_30_O_14_, as deduced from HREIMS peak at *m*/*z* 639.1721 [M + H]^+^. The ^1^H and ^13^C NMR spectra of **1** indicated four methyls (including two methoxyls); two methylenes; nine sp^3^ methines (including two oxygenated methines and five aromatic methines); and 17 nonprotonated carbons (including two ester carbonyls, two conjugated ketone carbonyls), which were similar to the tetrahydroxanthone dimers we reported before in Reference [2]. Careful inspection of 1D NMR spectra revealed the twin resonances indicating a dimeric structure for **1**, which was quite similar to that of asperdichrome, with obvious differences only in chemical shifts and coupling constants of H-5, H-6, and H-7 (*δ*_H-5_: 3.95 d (11.3) in **1** vs. 4.12 d (1.4) in asperdichrome; *δ*_H-6_: 2.42 m in **1** vs. 2.14 m in asperdichrome; and *δ*_H-7_: 2.72 dd (6.4, 10.6); 2.29 dd (11.0, 15.4) in **1** vs. 2.40, dd (18.8, 6.3); 2.52, dd (18.8, 11.6) in asperdichrome) (Figure 1, Table 1 and Supplementary Materials Figures S1–S21) [5]. Further interpretation of 2D NMR spectra confirmed compound **1** shared an identical planar structure with asperdichrome; however, it possessed different stereochemistry (Figure 2, Table 1 and Table 2). 

**Figure 3:**
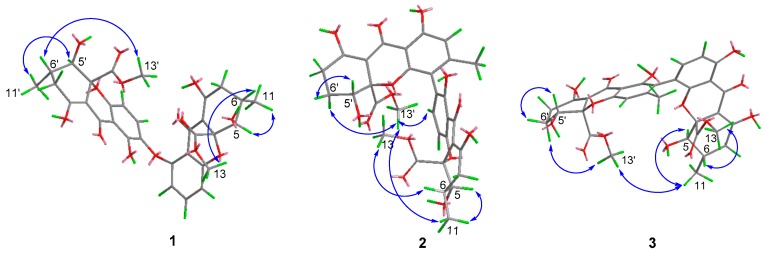
Key NOE correlations of **1**–**3**.

**Figure 4:**
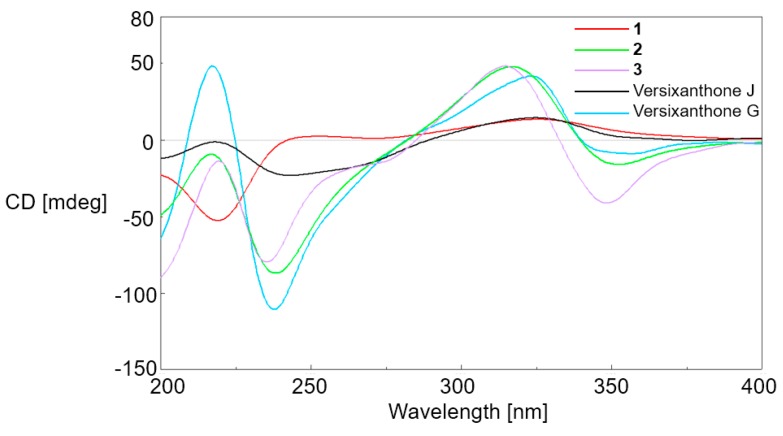
Circular dichroism (CD) spectra of **1**–**3**, versixanthones J and G.

The relative configuration of **1** was established as 5′R*, 6′S*, 10a′R*, 5R*, 6S*, 10aR* by the NOE correlations of Me-13 with H-6; H-5 with Me-11; Me-13′ with H-6′; and H-5′ with Me-11′, together with the coupling constant (^3^*J*_H-5_, _H-6_ = ^3^*J*_H-5′_, _H-6′_ = 11.3 Hz). This suggested that 13-COOCH_3_ and OH-5 were located at the same side of ring A, whilst Me-11 was located at the other side; similarly, 13′-COOCH_3_ and OH-5′ were located at the same side of ring F, whilst Me-11′ was located at the other side (Figure 3). The absolute configuration of **1** was determined by CD comparison. As there was no axial chirality between two monomeric units, the ECD curves were dominated by the chirality center elements [2,5,7]. Similar to versixanthones J, which we previously discovered from this strain, the positive n→π* CD band at around 330 nm, indicated the *R*-configuration at C-10a/C-10a′ (Figure 4) [2,5,7]. Combined with the relative configuration, the absolute configuration of compound **1** was assigned as 5′*R*, 6′*S*, 10a′*R*, 5*R*, 6*S*, 10a*R* and proved to be the 5-*epi* isomer of asperdichrome, and we named it 5-*epi*-asperdichrome.

Compounds **2** and **3** were also isolated as pale, yellow powder, and had the same molecular formula of C_32_H_30_O_14_, determined by HRESIMS detected at *m*/*z* 639.1721 [M + H]^+^. The almost twin resonances in the 1D NMR spectra, suggested that they were heterodimers of tetrahydroxanthones. A careful comparison of the NMR data of compounds **2** and **3** with versixanthone G, which we reported previously [2], suggested they had the same monomeric units. Further analysis of the 2D NMR data, revealed that the only difference between compounds **2**, **3** and versixanthone G were the linkage patterns of the two monomeric units. Compound **2** was constructed by connecting the two monomeric units via the linkage of C-2 and C-4′, based on the HMBC correlations from H-2′ to C-1′ and C-9a′; OH-1′ to C-1′; C-2′ and C-9a′; H_3_-11′ to C-2′; C-3′ and C-4′; OH-1 to C-1; C-2 and C-9a; and H-3 to C-4′. Based on the HMBC correlations from H-4′ to C-4a′ and C-3′; OH-1′ to C-1′; C-2′ and C-9a′; H_3_-11′ to C-2′; C-3′ and C-4′; OH-1 to C-1; C-2 and C-9a; and H-3 to C-2′ (Figure 2), the planar structure of compound **3** was completed by connecting the two monomeric units via the linkage between C-4 and C-2′, which was the same as purpureone, whose absolute configuration has not been reported [8,9]. 

The relative configurations of monomeric units in compounds **2** and **3**, were established to be the same as that of versixanthone G (5R*, 6S*, 10aR*, 5′R*, 10a′R*) by the coupling constant (^3^*J*_H-5_, _H-6_ = 11.1 Hz), and the NOE correlations between Me-13 and H-6, H-5 and Me-11, Me-13′ and H-6′a (*δ*_H_ 2.12 for **2** and *δ*_H_ 2.23 for **3**), H-5′ and H-6′b (*δ*_H_ 2.00 for **2** and *δ*_H_ 2.10 for **3**) (Figure 3, Table 1). Similar linkage type and substitution patterns with versixanthone G, suggest compounds **2** and **3** should also possess axial chirality [2]. The axial chiralities were further deduced by ECD exciton chirality method. Based on ECD spectra, the negative exciton couplings near 230 nm and 330 nm for **2** and **3**, which were similar with versixanthone G, allowing the assignment of both axial chiralities as a*R* [2]. Combined with the NOE correlations between two monomeric units (Me-13′ with H-3 and H-11 in **2**; Me-13′ with H-11 in **3**), the configurations at C-10a′ in both compounds were determined as *R*, and the absolute configurations of **2** and **3** were a*R*, 5*R*, 6*S*, 10a*R*, 5′*R*, 10a′*R,* accordingly. 

Biological evaluation of all the compounds by an MTT method showed that **2** and **3** exhibited extensive cytotoxicity against five kinds of cancer cell lines (HL-60, K562, H1975, MGC803, and HO-8910) (Table 3), with IC_50_ values ranging from 1.7 μM to 16.1 μM. The results suggested that the connection type between the two monomers was very important. As with connection by oxygen atoms, the cytotoxic activity disappears completely. The antimicrobial activities of compound **1** were also evaluated and showed mild activities against *Vibrio parahemolyticus*, *Bacillus subtilis*, *Mycobacterium Phlei*, and *Pseudomonas aeruginosa*, with MIC values ranging from 100 μM to 200 μM (Table 4). 

## 3. Materials and Methods 

### 3.1. General Experimental Procedures

UV spectra were measured on a Beckman DU 640 spectrophotometer (Beckman Coulter Inc., Brea, CA, USA). Specific rotations were obtained by a JASCO P-1020 digital polarimeter (JASCO Corporation, Tokyo, Japan). ESIMS were obtained on a Thermo Scientific LTQ Orbitrap XL mass spectrometer (Thermo Fisher Scientific, Waltham, MA, USA) or Micromass Q-TOF ULTIMA GLOBAL GAA076 LC Mass spectrometer (Wasters Corporation, Milford, MA, USA). NMR spectra were recorded on an Agilent 500 MHz DD2 spectrometer (Agilent Technologies Inc., Santa Clara, CA, USA), using TMS as an internal standard and chemical shifts were recorded as *δ*-values. Semi-preparative HPLC employed an ODS column (HPLC (YMC-Pack ODS-A, 10 × 250 mm, 5 μm, 3 mL/min)) (YMC Co., Ltd., Kyoto, Japan). Medium-pressure preparation liquid chromatography (MPLC) was performed on a Bona-Agela CHEETAHTM HP100 (Beijing Agela Technologies Co., Ltd., Beijing, China) [10]. 

### 3.2. Fungal Material

As described previously in Reference [6]. 

### 3.3. Fermentation and Extraction 

As described previously in Reference [6]. 

### 3.4. Isolation 

The organic extract was subjected to vacuum liquid chromatography over a silica gel column, using a gradient elution with petroleum ether-CH_2_Cl_2_-MeOH to give six fractions (fractions 1–6). Guided by LC-MS-UV data, fraction 3 (6.5 g) eluted with CH_2_Cl_2_-MeOH (97:3) was applied on a C-18 ODS column using a stepped gradient elution of MeOH-H_2_O, yielding five subfractions (fractions 3.1–3.5). Fraction 3.4, which eluted with MeOH-H_2_O (75:25) was chromatographed on SephadexLH-20 with CH_2_Cl_2_-MeOH (1:1), and further separated by MPLC (C-18 ODS) using MeOH-H_2_O (a gradient elution, 30–100%), to furnish five subfractions (fractions 3.4.1–3.4.5). Fractions 3.4.1 was purified by semi-preparative HPLC (MeOH-H_2_O (55:45)), to afford compound **1** (4.0 mg, *t*_R_ 18.5 min). Fraction 3.4.5 was also purified by semi-preparative HPLC, first using a gradient elution by MeOH-H_2_O (30–100%), and then MeCN-H_2_O (50–90%) to afford compounds **2** (3.0 mg, *t*_R_ 22.5 min) and **3** (4.0 mg, *t*_R_ 25.0 min).

**5-*epi*-asperdichrome (1):** pale yellow powder; ${\left[\alpha \right]}_{D}^{20}$ −6.6 (*c* 0.1, CHCl_3_); ECD (MeOH) λ (nm) (Δε): 326 (+12.5), 273 (+0.4), 249 (+2.5), 220 (−53.5); UV (MeOH) *λ*_max_ (log *ε*): 242 (2.10), 318 (3.46) nm; ^1^H-NMR and ^13^C-NMR (see Table 1 and Table 2); HR-ESI-MS [M + H]^+^ *m*/*z* 639.1721 (calcd. for C_32_H_31_O_14_, 639.1708, error = 1.9432 ppm), [M + Na] *m*/*z* 661.1538 (calcd. for C_32_H_30_O_14_Na, 661.1528, error = 1.6056 ppm).

**Versicolone N (2):** pale yellow powder; ${\left[\alpha \right]}_{D}^{20}$ −43.0 (*c* 0.1, CHCl_3_); ECD (MeOH) λ (nm) (Δε): 360 (−16.5), 318 (+49.7), 237 (−91.5), 218 (−3.5); UV (MeOH) *λ*_max_ (log *ε*): 238 (2.30), 313 (3.16) nm; ^1^H-NMR and ^13^C-NMR (see Table 1 and Table 2); HR-ESI-MS [M + H]^+^ *m*/*z* 639.1702 (calcd. for C_32_H_31_O_14_, 639.1708, error = −1.0639 ppm), [M + NH_4_] *m*/*z* 656.1976 (calcd. for C_32_H_34_O_14_N, 656.1974, error = 0.2826 ppm).

**Versicolone O (3):** pale yellow powder; ${\left[\alpha \right]}_{D}^{20}$ −114.8 (*c* 0.1, CHCl_3_); ECD (MeOH) λ (nm) (Δε): 349 (−41.5), 315 (+48.0), 235 (−80.7), 220 (−13.0); UV (MeOH) *λ*_max_ (log *ε*): 240 (2.25), 315 (3.26) nm; ^1^H-NMR and ^13^C-NMR (see Table 1 and Table 2); HR-ESI-MS [M + H]^+^ *m*/*z* 639.1703 (calcd. for C_32_H_31_O_14_, 639.1708, error = −0.7571 ppm), [M + Na] *m*/*z* 661.1522 (calcd. for C_32_H_30_O_14_Na, 661.1528, error = −0.8306 ppm). 

### 3.5. Assay of Cytotoxicity

These biological evaluations were carried out as previously reported in Reference [11]. 

### 3.6. Assay of Antimicrobial Activity

The microorganism suspension (198 μL, 10^6^ cfu/mL) in autoclaved growth medium (peptone (20 g/L), beef extract (3 g/L), NaCl (5 g/L) for bacteria; peptone (20 g/L), yeast extract (10 g/L), glucose (20 g/L) for *Candida albicans*)) was added to each well of 96-well plates. Solutions (40 mM) of the compounds and positive drugs were made up in DMSO and dispensed into 96-well plates using the 2× microdilution method, to give 16 concentrations in the range of 200–0.006 μM. Incubated at 28 °C for 24 h, and the lowest concentration that gave complete growth inhibition was recorded as the minimum inhibitory concentration (MIC). 

## 4. Conclusions

In summary, UPLC-MS guided chemical investigation led to the discovery of three new tetrahydroxanthone dimers, 5-*epi*-asperdichrome (**1**), versixanthones N (**2**), and O (**3**), from the marine-derived fungus *Aspergillus versicolor* HDN1009. Compound **1** represented the second example of tetrahydroxanthone dimers with a diaryl ether bond linkage. Their absolute configurations, including the axial and central chirality elements were elucidated by NOE experiments and circular dichroism (CD) comparison. Compounds **2** and **3,** exhibited extensive cytotoxicities against five cancer cell lines with IC_50_ values ranging from 1.7 μM to 16.1 μM, whilst compound **1** showed mild activities against four kinds of bacterial strains, with MIC values ranging from 100 μM to 200 μM.

## Figures and Tables

**Table 1:** ^1^H NMR data of compounds **1**–**3** (500 MHz, CDCl_3_, TMS, *δ* ppm, *J* in Hz).

No.	1	Asperdichrome	2	3
2	6.36 d (8.4)	6.39, d (8.2)	--	6.62 d (8.4)
3	7.30 t (8.3)	7.28, t (8.2)	7.69 d (8.4)	7.18 d (8.4)
4	6.80 d (8.4)	6.76, d (8.2)	6.69 d (8.4)	--
5	3.95 d (11.3)	4.12 d (1.4)	3.94 d (11.1)	3.82 d (11.1)
6	2.42 m	2.14, m	2.46 m	2.34 m
7	2.72 dd (6.4, 10.6) 2.29 dd (11.0, 15.4)	2.52, dd (18.8, 11.6) 2.40, dd (18.8, 6.3)	2.74 dd (6.3, 19.3) 2.32 dd (10.7, 19.2)	2.69 m
11	1.17 d (5.9)	1.18, d (6.3)	1.18 d (6.4)	1.08 d (6.3)
13	3.72 s	3.69 s	3.70 s	3.66
1-OH	--		11.49 s	11.41 s
8-OH	15.78 s	16.0, s	13.75 brs	13.68 brs
2′	--	--	6.51 s	--
3′	7.22 d (8.9)	7.22, d (8.7)	--	--
4′	6.57 d (8.9)	6.57, d (8.7)	--	6.56 s
5′	3.94 d (11.3)	3.94, d (11.1)	4.14 dd (5.0, 12.6)	4.33 dd (5.0, 12.4)
6′	2.43 m	2.40, m	2.12 m; 2.00, m	2.23 m; 2.10, m
7′	2.76 dd (6.1, 10.4); 2.32 dd (4.1, 8.2)	2.75, dd (18.7, 5.8) 2.32, dd (18.7, 10.9)	2.62 m	2.66 m
11′	1.18 d (5.7)	1.17, d (6.8)	2.12 s	2.23 s
13′	3.70 s	3.72, s	3.74 s	3.73 s
1′-OH	11.35 s	11.4, s	11.25 brs	11.44 brs
8′-OH	13.75 s	13.7, s	13.77 brs	13.86 brs

**Table 2:** ^13^C NMR data of compounds **1**–**3** in CDCl_3_ (125 MHz, CDCl_3_, TMS, *δ* ppm).

No.	1	Asperdi- chrome	2	3
1	158.7	160.0	159.3	161.9
2	109.5	110.9	117.1	110.5
3	135.6	136.6	141.0	140.2
4	111.9	113.0	107.8	114.7
4a	160.2	160.8	158.5	155.9
5	77.9	72.4	76.9	76.6
6	29.2	30.1	29.3	29.2
7	37.8	35.1	36.2	36.1
8	182.5	186.4	177.7	177.3
8a	102.7	103.2	101.6	101.4
9	180.2	181.7	187.1	187.2
9a	110.8	112.0	106.9	107.0
10a	84.9	86.2	85.0	84.7
11	18.0	17.9	18.0	17.9
12	170.1	173.5	170.5	169.9
13	53.0	53.7	53.2	53.0
1′	158.7	154.1	159.3	161.9
2′	136.8	137.8	111.8	118.4
3′	130.3	131.5	150.2	149.8
4′	107.6	109.1	115.7	109.1
4a′	155.5	157.9	155.7	157.8
5′	77.9	77.6	71.5	71.9
6′	29.3	31.1	23.5	23.9
7′	36.3	37.1	27.5	27.6
8′	178.3	179.6	177.3	177.8
8a′	101.6	103.3	101.1	101.3
9′	186.9	188.7	186.8	186.7
9a′	107.9	109.1	105.1	104.5
10a′	84.9	86.8	84.7	84.5
11′	18.0	18.3	21.1	21.5
12′	170.2	172.1	169.8	170.4
13′	53.2	53.4	53.1	53.2

**Table 3:** Inhibitory effects of **1**–**3** on six tumor cells.

Comp.	IC_50_ (μM)					
	MGC803	HL-60	HO-8910	H1975	K562	A549
1	>30	>30	>30	>30	>30	>30
2	1.7	2.7	8.5	8.8	9.1	>30
3	1.8	8.1	6.7	8.5	16.1	>30
Dox ^a^	0.2	0.02	0.5	0.8	0.3	0.2

^a^ Dox stands for doxorubicin hydrochloride, which was used as a reference drug.

**Table 4:** Inhibitory effects of **1** on six kinds of microorganisms.

Comp.	MIC (μM)					
	*Vibrio parahemolyticus*	*Bacillus subtilis*	*Mycobacterium phlei*	*Escherichia coli*	*Pseudomonas aeruginosa*	*Candida albicans*
1	100	200	200	>200	100	>200
Positive Drug	0.781 ^a^	0.391 ^a^	0.781 ^a^	0.391 ^a^	1.56 ^a^	3.13 ^b^

^a^ Ciprofloxacin used as a reference drug for bacteria; ^b^ Nystatin used as a reference drug for *Candida albicans*.

## Supplementary Materials

The following are available online at http://www.mdpi.com/1660-3397/16/9/335/s1, Figures S1–S7: 1D, 2D NMR and HRESIMS spectra of 5-*epi*-asperdichrome (**1**), Figures S8–S14: 1D, 2D NMR and HRESIMS spectra of versixanthone N (**2**), Figures S15–S21: 1D, 2D NMR and HRESIMS spectra of versixanthone O (**3**).
