# Supplementary material for: Cytotoxic Tetrahydroxanthone Dimers from the Mangrove-Associated Fungus Aspergillus versicolor HDN1009
Source: Mar Drugs. 2018 Sep 14;16(9):335. doi: 10.3390/md16090335 (PMC6164687; doi:10.3390/md16090335)

Supporting Information

Cytotoxic Tetrahydroxanthone Dimers from the Mangrove-Associated Fungus *Aspergillus versicolor* HDN1009

Guihong Yu [1], Guangwei Wu [1], Zichao Sun [1], Xiaomin Zhang [1], Qian Che [1], Qianqun Gu [1], Tianjiao, Zhu [1,2], Dehai Li [1,2,3] and Guojian Zhang[1,2,]*

[1] Key Laboratory of Marine Drugs, Chinese Ministry of Education, School of Medicine and Pharmacy, Ocean University of China, Qingdao 266003, P. R. China; Yuguihong1990@126.com (G.Y.); gweiwu@163.com (G.W.); 171774170@163.com (Z.S.); xiaominzhang91@163.com (X.Z.); cheqian064@ouc.edu.cn (Q. C.); guqianq@ouc.edu.cn (Q.G.) zhutj@ouc.edu.cn (T.Z.); dehaili@ouc.edu.cn (D.L.)

[2] Laboratory for Marine Drugs and Bioproducts of Qingdao Pilot National Laboratory for Marine Science and Technology, Qingdao, 266237, P. R. China

[3] Open Studio for Druggability Research of Marine Natural Products, Qingdao Pilot National Laboratory for Marine Science and Technology, Qingdao, 266237, China

* Correspondence: E-mail: zhangguojian@ouc.edu.cn; Tel.: +86 532-82032065.

List of Supporting Information

Figure S1. ^{1}H NMR spectrum (500 MHz) of 5-*epi*-asperdichrome (**1**) in $CDCl_3$.............S3

Figure S2. ^{13}C NMR spectrum (125 MHz) of 5-*epi*-asperdichrome (**1**) in $CDCl_3$............S3

Figure S3. HSQC spectrum of 5-*epi*-asperdichrome (**1**) S4

Figure S4. ^{1}H-^{1}H COSY spectrum of 5-*epi*-asperdichrome (**1**) S4

Figure S5. HMBC spectrum of 5-*epi*-asperdichrome (**1**) S5

Figure S6. NOE spectrum of 5-*epi*-asperdichrome (**1**) S5

Figure S7. HRESIMS spectrum of 5-*epi*-asperdichrome (**1**) S7

Figure S8. ^{1}H NMR spectrum (500 MHz) of versixanthone N (**2**) in $CDCl_3$...............S8

Figure S9. ^{13}C NMR spectrum (125 MHz) of versixanthone N (**2**) in $CDCl_3$………..S8

Figure S10. HSQC spectrum of versixanthone N (**2**) S9

Figure S11. ^{1}H-^{1}H COSY spectrum of versixanthone N (**2**) S9

Figure S12. HMBC spectrum of versixanthone N (**2**) S10

Figure S13. NOE spectrum of versixanthone N (**2**) S10

Figure S14. HRESIMS spectrum of versixanthone N (**2**) S13

Figure S15. ^{1}H NMR spectrum (500 MHz) of versixanthone O (**3**) in $CDCl_3$.............S13

Figure S16. ^{13}C NMR spectrum (125 MHz) of versixanthone O (**3**) in $CDCl_3$............S14

Figure S17. HSQC spectrum of versixanthone O (**3**) S14

Figure S18. ^{1}H-^{1}H COSY spectrum of versixanthone O (**3**)..S15

Figure S19. HMBC spectrum of versixanthone O (**3**) S15

Figure S20. NOE spectrum of versixanthone O (**3**) S16

Figure S21. HRESIMS spectrum of versixanthone O (**3**) S18

Figure S1. ^{1}H NMR spectrum (500 MHz) of 5-*epi*-asperdichrome (**1**) in $CDCl_3$

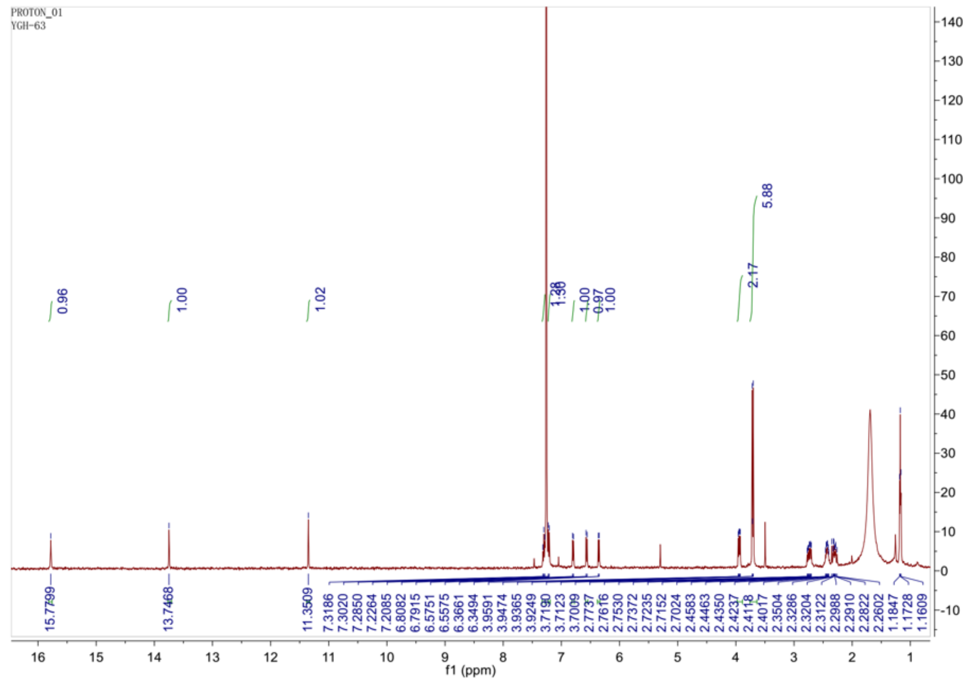

No.	Parameter	Value
1	Origin	Varian
2	Spectrometer	vnmrs
3	Solvent	cdcl3
4	Volume of deuterated solvent	200 µL
5	Internal standard	TMS
6	Temperature	25.0
7	Pulse Sequence	s2pul
8	Number of Scans	40
9	Experiment	1D
10	Probe	OneP
11	Receiver Gain	50
12	Relaxation Delay	1.0000
13	Pulse Width	0.0000
14	Acquisition Time	2.0447
15	Spectrometer Frequency	499.79
16	Spectral Width	8012.8
17	Lowest Frequency	-1007.7
18	Nucleus	1H
19	Acquired Size	16384
20	Spectral Size	32768

Figure S2. ^{13}C NMR spectrum (125 MHz) of 5-*epi*-asperdichrome (**1**) in $CDCl_3$

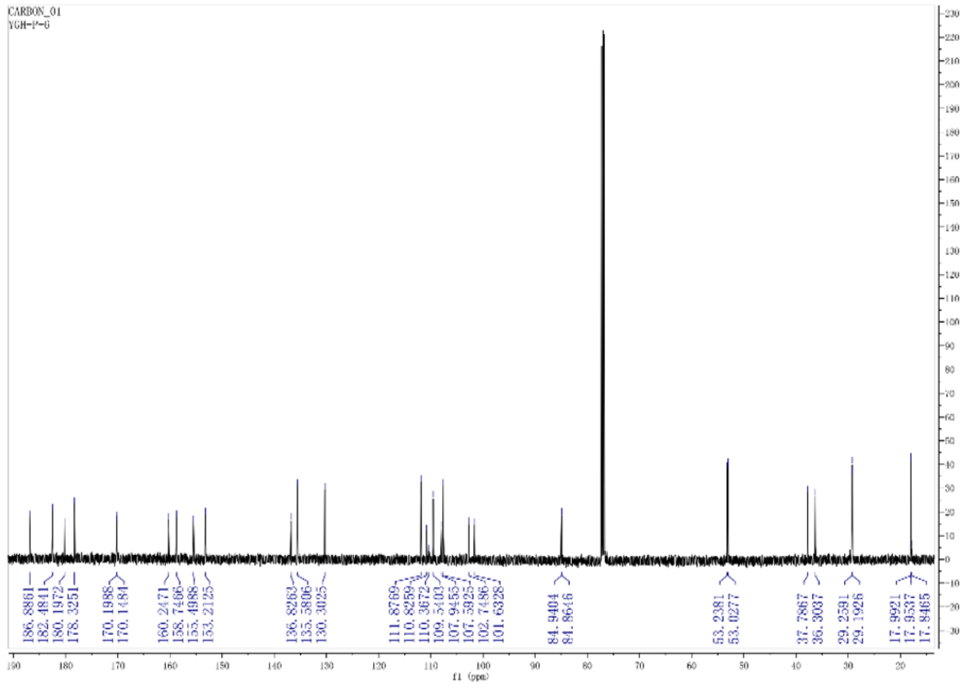

No.	Parameter	Value
1	Origin	Varian
2	Spectrometer	vnmrs
3	Solvent	cdcl3
4	Volume of deuterated solvent	200 µL
5	Internal standard	TMS
6	Temperature	25.0
7	Pulse Sequence	s2pul
8	Experiment	1D
9	Probe	OneP
10	Number of Scans	10000
11	Receiver Gain	30
12	Relaxation Delay	1.0000
13	Pulse Width	0.0000
14	Acquisition Time	1.0486
15	Spectrometer Frequency	125.68
16	Spectral Width	31250.0
17	Lowest Frequency	-1801.2
18	Nucleus	13C
19	Acquired Size	32768
20	Spectral Size	65536

Figure S3. HSQC spectrum of 5-*epi*-asperdichrome (**1**)

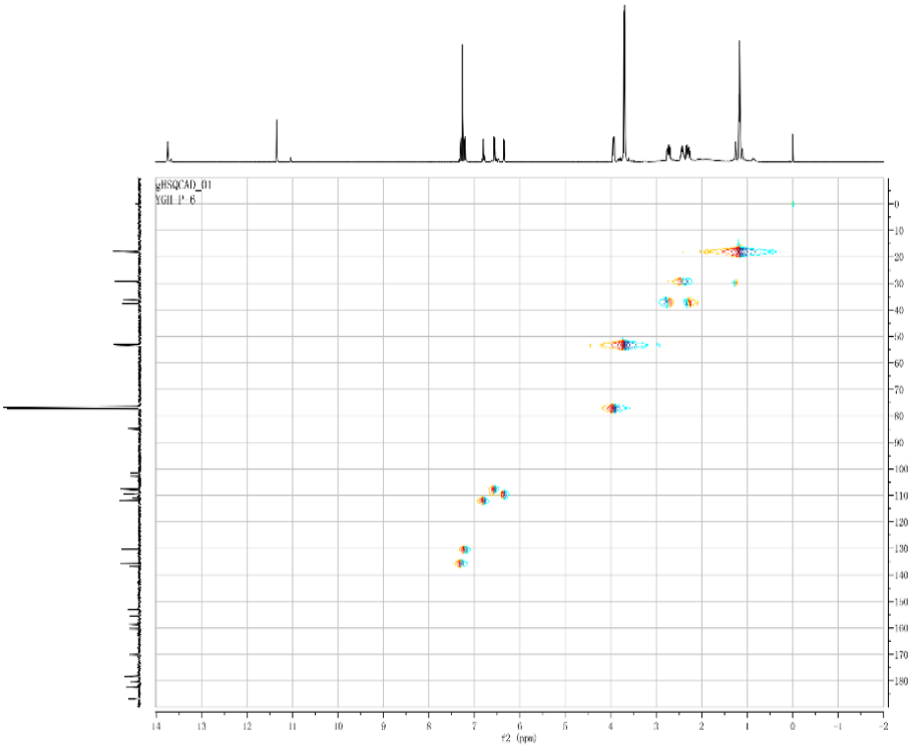

No.	Parameter	Value
1	Origin	Varian
2	Spectrometer	vnmrs
3	Solvent	cdcl3
4	Volume of deuterated solvent	200 µL
5	Internal standard	TMS
6	Temperature	25.0
7	Pulse Sequence	gHSQCAD
8	Experiment	HSQC-EDITED
9	Probe	OneP
10	Number of Scans	32
11	Receiver Gain	30
12	Relaxation Delay	1.0000
13	Pulse Width	0.0000
14	Acquisition Time	0.1500
15	Spectrometer Frequency	(499.79, 125.68)
16	Spectral Width	(8012.8, 25133.5)
17	Lowest Frequency	(-1007.7, -1256.4)
18	Nucleus	(1H, 13C)
19	Acquired Size	(1202, 96)
20	Spectral Size	(2048, 1024)

Figure S4. ^{1}H-^{1}H COSY spectrum of 5-*epi*-asperdichrome (**1**)

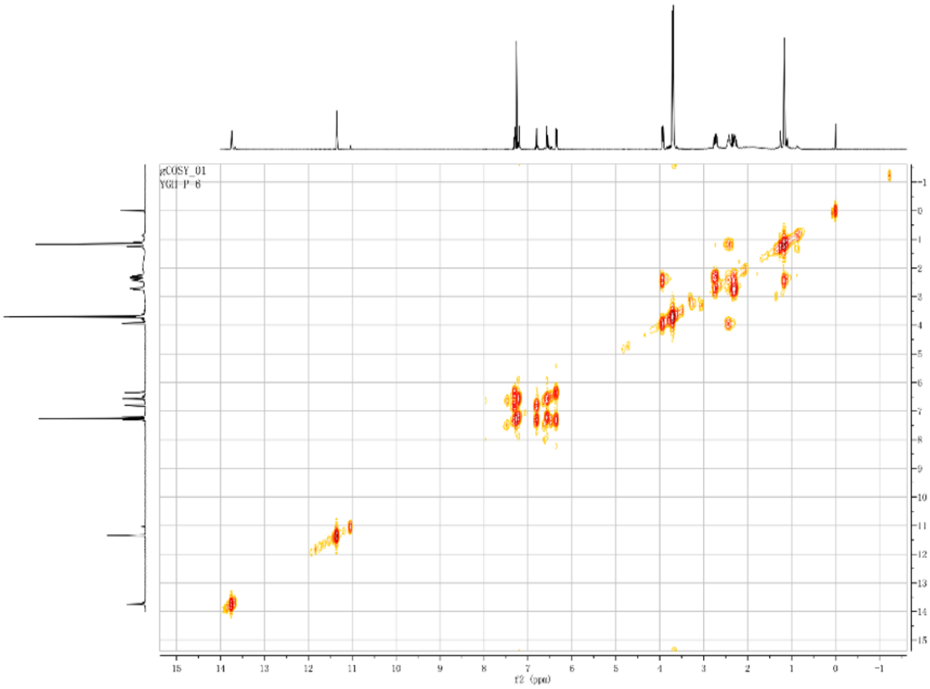

No.	Parameter	Value
1	Origin	Varian
2	Spectrometer	vnmrs
3	Solvent	cdcl3
4	Volume of deuterated solvent	200 µL
5	Internal standard	TMS
6	Temperature	25
7	Pulse Sequence	gCOSY
8	Experiment	COSY
9	Probe	OneP
10	Number of Scans	16
11	Receiver Gain	52
12	Relaxation Delay	1
13	Pulse Width	0
14	Acquisition Time	0.1501
15	Spectrometer Frequency	(499.79, 499.79)
16	Spectral Width	(8503.4, 8503.4)
17	Lowest Frequency	(-816.7, -816.7)
18	Nucleus	(1H, 1H)
19	Acquired Size	(1276, 128)
20	Spectral Size	(2048, 2048)

Figure S5. HMBC spectrum of 5-*epi*-asperdichrome (**1**)

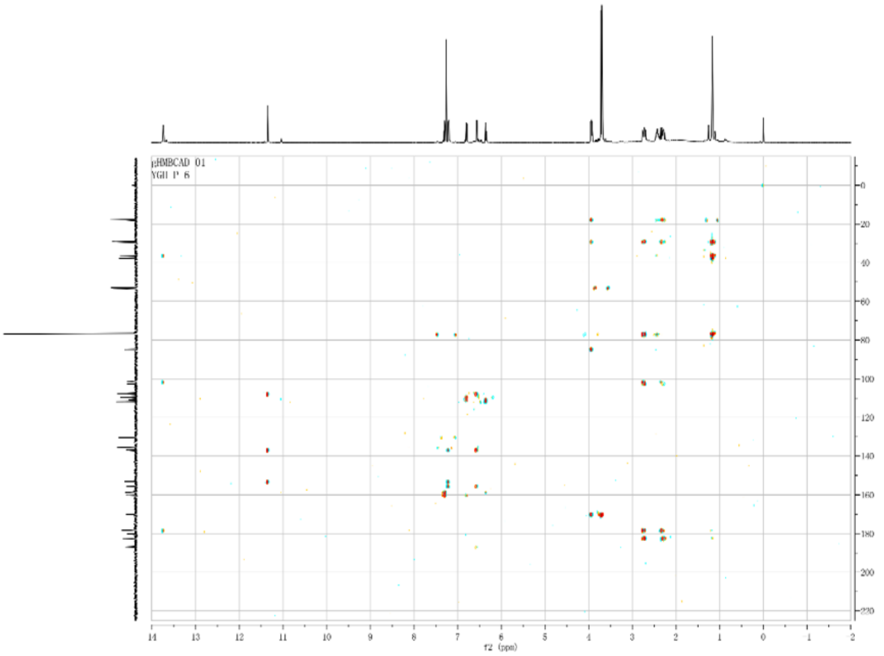

No.	Parameter	Value (f2, f1)
1	Origin	Varian
2	Spectrometer	vnmrs
3	Solvent	cdcl3
4	Volume of deuterated solvent	200 μL
5	Internal standard	TMS
6	Temperature	25
7	Pulse Sequence	gHMBCAD
8	Experiment	HMBC
9	Probe	OneP
10	Number of Scans	30
11	Receiver Gain	30
12	Relaxation Delay	1
13	Pulse Width	0
14	Acquisition Time	0.15
15	Spectrometer Frequency	(499.79, 125.68)
16	Spectral Width	(8012.8, 30165.9)
17	Lowest Frequency	(-1007.7, -1887.6)
18	Nucleus	(1H, 13C)
19	Acquired Size	(1202, 200)
20	Spectral Size	(2048, 2048)

Figure S6. NOE spectrum of 5-*epi*-asperdichrome (**1**)

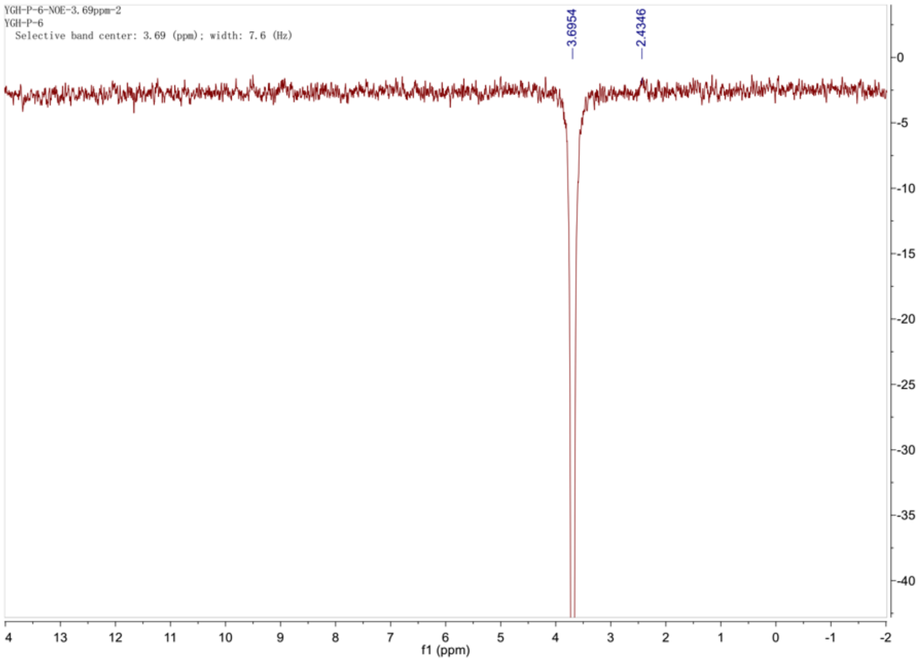

No.	Parameter	Value
1	Origin	Varian
2	Instrument	vnmrs
3	Solvent	cdcl3
4	Volume of deuterated solvent	200 μL
5	Internal standard	TMS
6	Temperature	25
7	Pulse Sequence	NOESY1D
8	Experiment	1D-NOESY
9	Probe	OneP
10	Number of Scans	2500
11	Receiver Gain	50
12	Relaxation Delay	1
13	Pulse Width	10.3
14	Acquisition Time	2.0447
15	Spectrometer Frequency	499.79
16	Spectral Width	8012.8
17	Lowest Frequency	-1007.7
18	Nucleus	1H
19	Acquired Size	16384
20	Spectral Size	65536

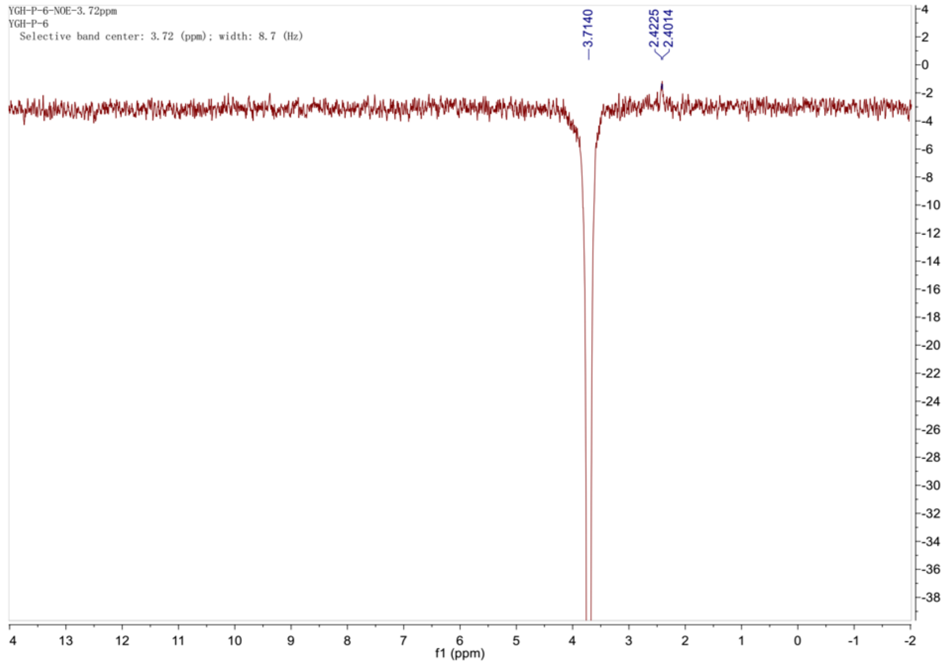

No.	Parameter	Value
1	Origin	Varian
2	Instrument	vnmrs
3	Solvent	cdcl3
4	Volume of deuterated solvent	200 µL
5	Internal standard	TMS
6	Temperature	25
7	Pulse Sequence	NOESY1D
8	Experiment	1D-NOESY
9	Probe	OneP
10	Number of Scans	2500
11	Receiver Gain	50
12	Relaxation Delay	1
13	Pulse Width	10.3
14	Acquisition Time	2.0447
15	Spectrometer Frequency	499.79
16	Spectral Width	8012.8
17	Lowest Frequency	-1007.7
18	Nucleus	1H
19	Acquired Size	16384
20	Spectral Size	65536

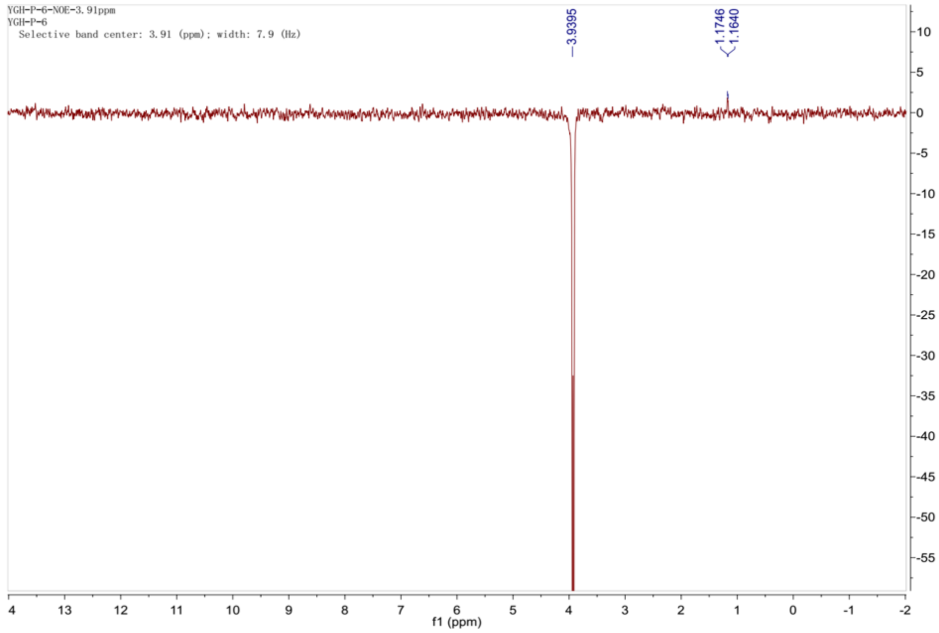

No.	Parameter	Value
1	Origin	Varian
2	Instrument	vnmrs
3	Solvent	cdcl3
4	Volume of deuterated solvent	200 µL
5	Internal standard	TMS
6	Temperature	25
7	Pulse Sequence	NOESY1D
8	Experiment	1D-NOESY
9	Probe	OneP
10	Number of Scans	2500
11	Receiver Gain	50
12	Relaxation Delay	1
13	Pulse Width	10.3
14	Acquisition Time	2.0447
15	Spectrometer Frequency	499.79
16	Spectral Width	8012.8
17	Lowest Frequency	-1007.7
18	Nucleus	1H
19	Acquired Size	16384
20	Spectral Size	65536

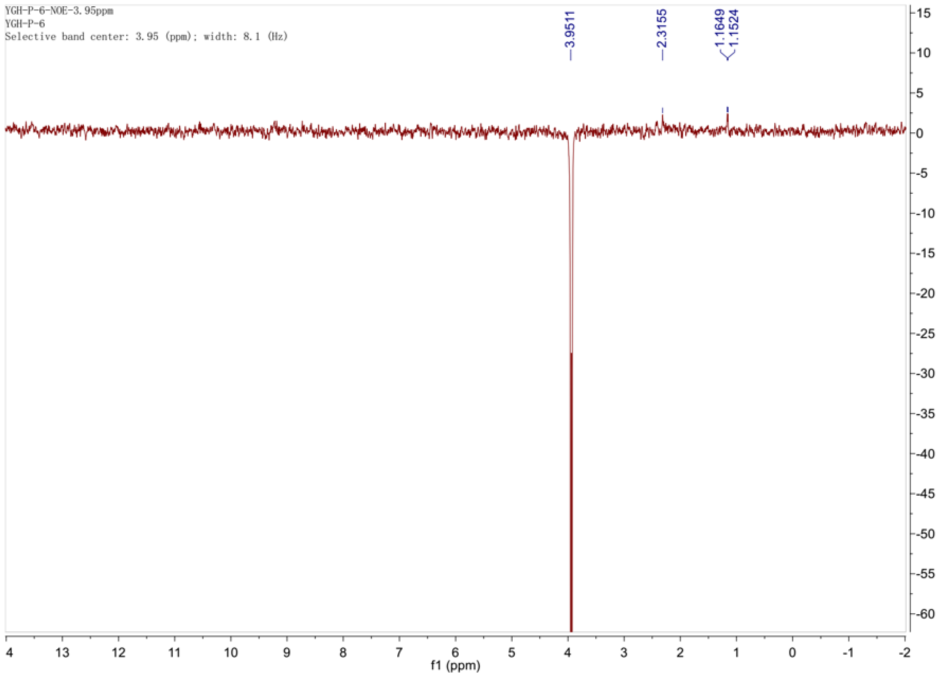

No.	Parameter	Value
1	Origin	Varian
2	Instrument	vnmrs
3	Solvent	cdcl3
4	Volume of deuterated solvent	200 µL
5	Internal standard	TMS
6	Temperature	25
7	Pulse Sequence	NOESY1D
8	Experiment	1D-NOESY
9	Probe	OneP
10	Number of Scans	2500
11	Receiver Gain	50
12	Relaxation Delay	1
13	Pulse Width	10.3
14	Acquisition Time	2.0447
15	Spectrometer Frequency	499.79
16	Spectral Width	8012.8
17	Lowest Frequency	-1007.7
18	Nucleus	1H
19	Acquired Size	16384
20	Spectral Size	65536

Figure S7. HRESIMS spectrum of 5-*epi*-asperdichrome (**1**)

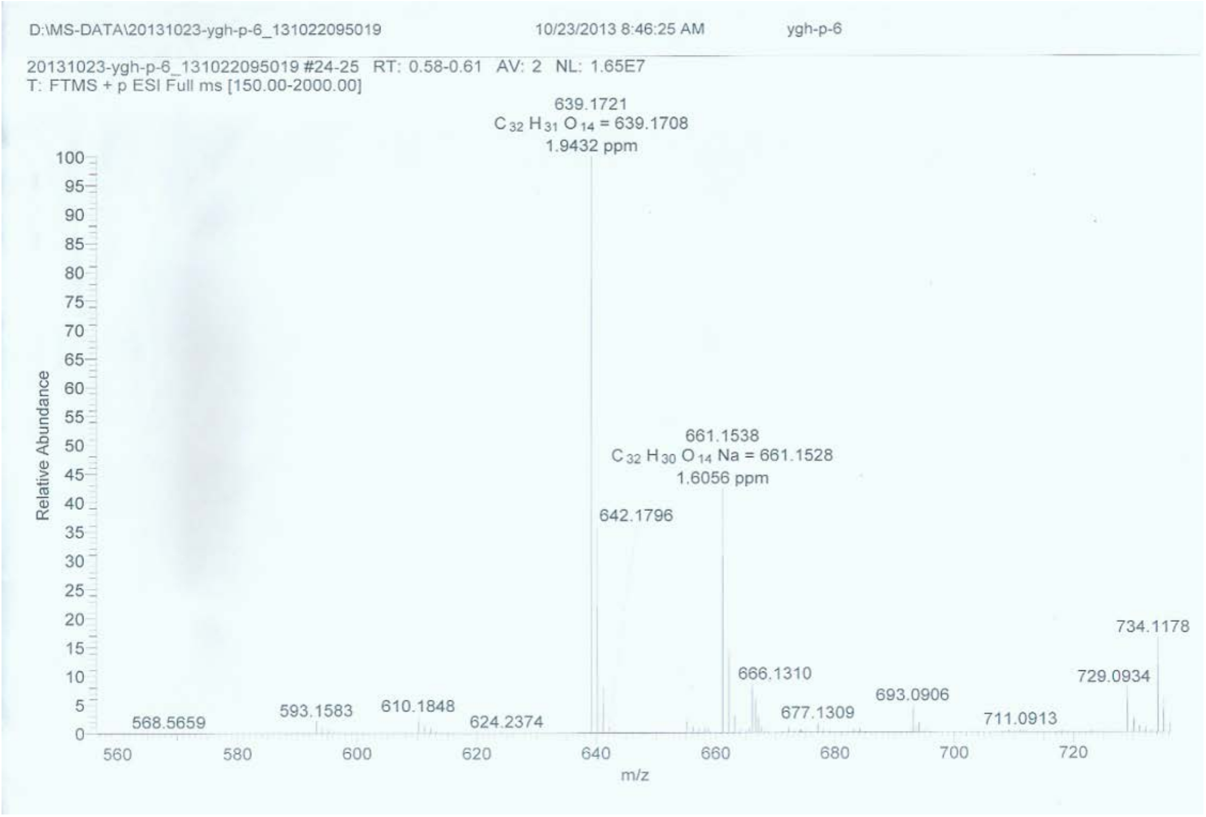

Figure S8. ^{1}H NMR spectrum (500 MHz) of versixanthone N (**2**) in $CDCl_3$

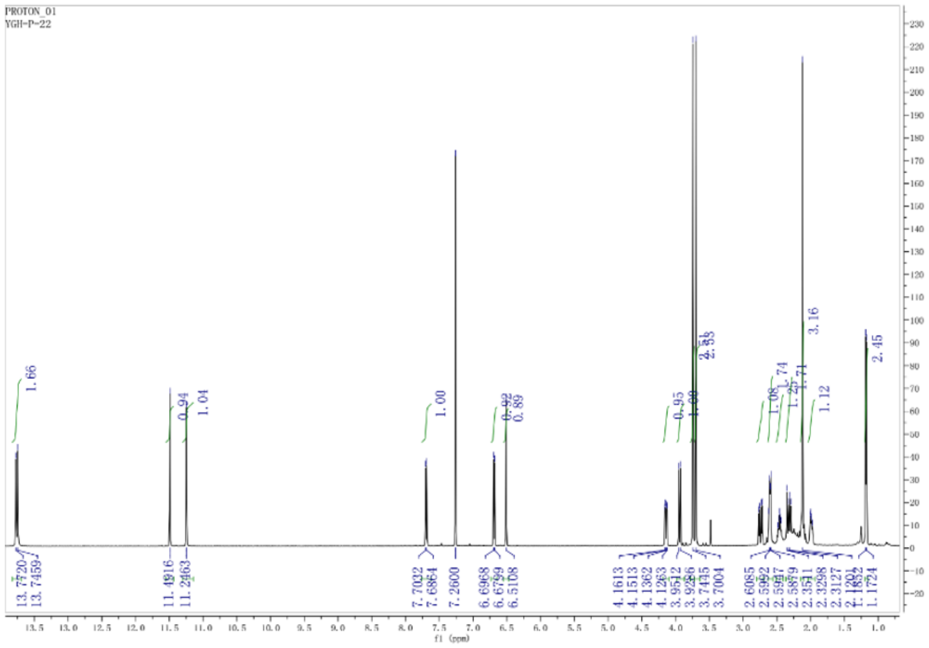

No.	Parameter	Value
1	Origin	Varian
2	Instrument	vnmrs
3	Solvent	cdcl3
4	Volume of deuterated solvent	500 µL
5	Internal standard	TMS
6	Temperature	25
7	Pulse Sequence	s2pul
8	Experiment	1D
9	Probe	OneP
10	Number of Scans	8
11	Receiver Gain	30
12	Relaxation Delay	1
13	Pulse Width	5.15
14	Acquisition Time	2.0447
15	Spectrometer Frequency	499.79
16	Spectral Width	8012.8
17	Lowest Frequency	-1007.7
18	Nucleus	1H
19	Acquired Size	16384
20	Spectral Size	65536

Figure S9. ^{13}C NMR spectrum (125 MHz) of versixanthone N (**2**) in $CDCl_3$

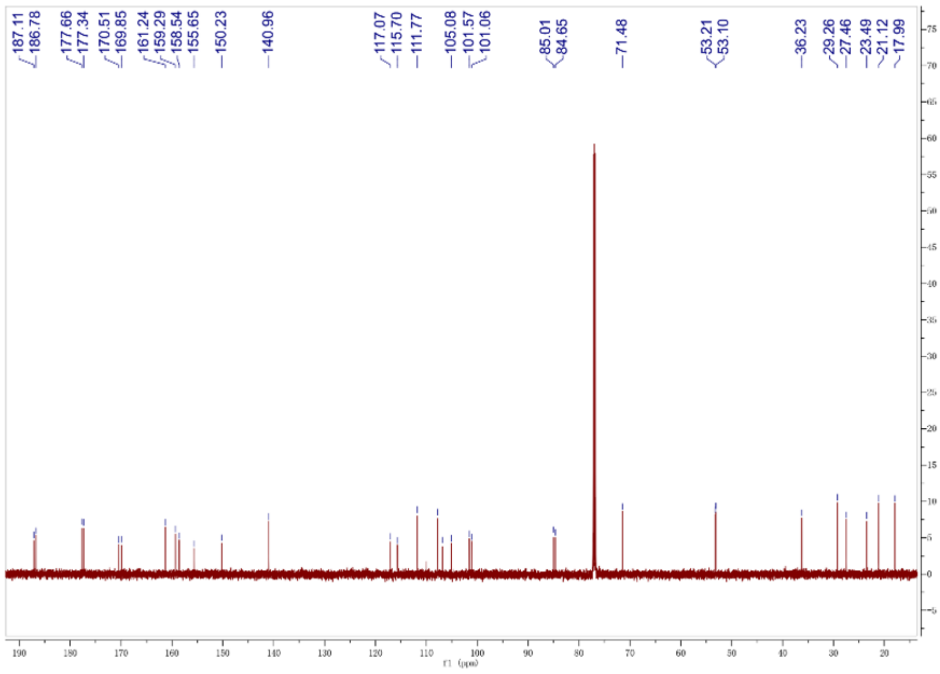

No.	Parameter	Value
1	Origin	Varian
2	Instrument	vnmrs
3	Solvent	cdcl3
4	Volume of deuterated solvent	500 µL
5	Internal standard	TMS
6	Temperature	25
7	Pulse Sequence	s2pul
8	Experiment	1D
9	Probe	OneP
10	Number of Scans	272
11	Receiver Gain	30
12	Relaxation Delay	1
13	Pulse Width	4.95
14	Acquisition Time	1.0486
15	Spectrometer Frequency	125.68
16	Spectral Width	31250
17	Lowest Frequency	-1801.2
18	Nucleus	13C
19	Acquired Size	32768
20	Spectral Size	65536

Figure S10. HSQC spectrum of versixanthone N (**2**)

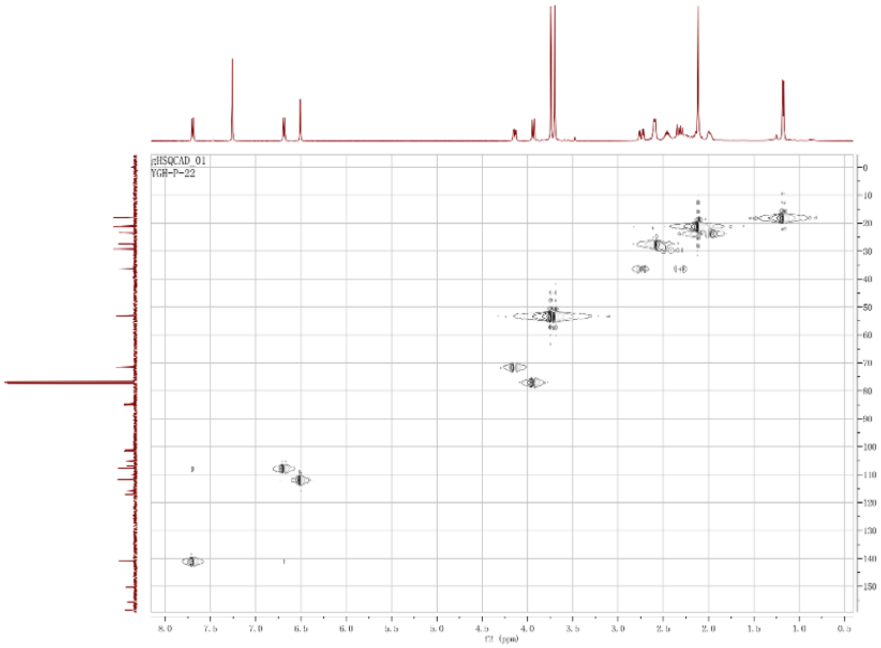

No.	Parameter	Value
1	Origin	Varian
2	Instrument	vnmrs
3	Solvent	cdcl3
4	Volume of deuterated solvent	500 µL
5	Internal standard	TMS
6	Temperature	25
7	Pulse Sequence	gHSQCAD
8	Experiment	HSQC-EDITED
9	Probe	OneP
10	Number of Scans	8
11	Receiver Gain	30
12	Relaxation Delay	1
13	Pulse Width	10.3
14	Acquisition Time	0.15
15	Spectrometer Frequency	(499.79, 125.68)
16	Spectral Width	(8012.8, 25133.5)
17	Lowest Frequency	(-1007.7, -1256.4)
18	Nucleus	(1H, 13C)
19	Acquired Size	(1202, 96)
20	Spectral Size	(2048, 1024)

Figure S11. ^{1}H-^{1}H COSY spectrum of versixanthone N (**2**)

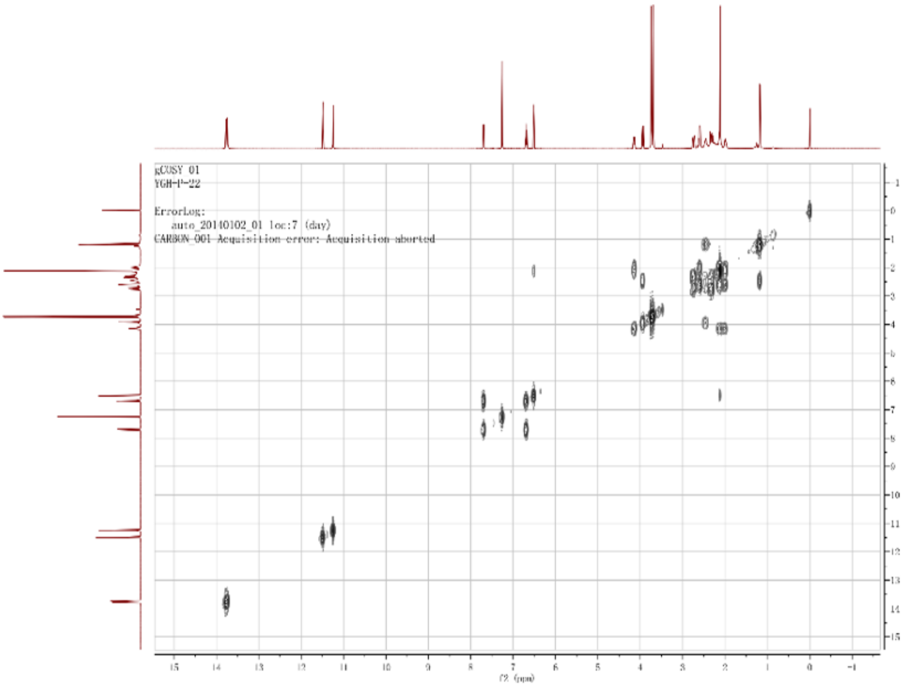

No.	Parameter	Value
1	Origin	Varian
2	Instrument	vnmrs
3	Solvent	cdcl3
4	Volume of deuterated solvent	500 µL
5	Internal standard	TMS
6	Temperature	25
7	Pulse Sequence	gCOSY
8	Experiment	COSY
9	Probe	OneP
10	Number of Scans	2
11	Receiver Gain	30
12	Relaxation Delay	1
13	Pulse Width	10.3
14	Acquisition Time	0.15
15	Spectrometer Frequency	(499.79, 499.79)
16	Spectral Width	(8561.6, 8561.6)
17	Lowest Frequency	(-835.6, -835.6)
18	Nucleus	(1H, 1H)
19	Acquired Size	(1284, 128)
20	Spectral Size	(2048, 2048)

Figure S12. HMBC spectrum of versixanthone N (**2**)

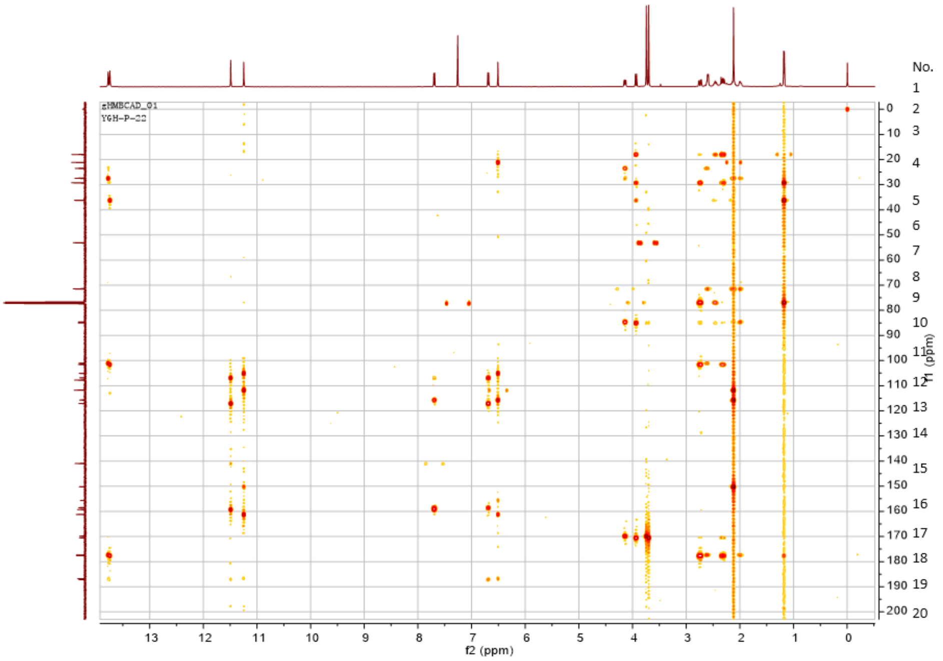

No.	Parameter	Value
1	Origin	Varian
2	Instrument	vnmrs
3	Solvent	cdcl3
4	Volume of deuterated solvent	500 µL
5	Internal standard	TMS
6	Temperature	25
7	Pulse Sequence	gHMBCAD
8	Experiment	HMBC
9	Probe	OneP
10	Number of Scans	6
11	Receiver Gain	30
12	Relaxation Delay	1
13	Pulse Width	10.3
14	Acquisition Time	0.15
15	Spectrometer Frequency	(499.79, 125.68)
16	Spectral Width	(8012.8, 30165.9)
17	Lowest Frequency	(-1007.7, -1887.6)
18	Nucleus	(1H, 13C)
19	Acquired Size	(1202, 200)
20	Spectral Size	(2048, 2048)

Figure S13. NOE spectrum of versixanthone N (**2**)

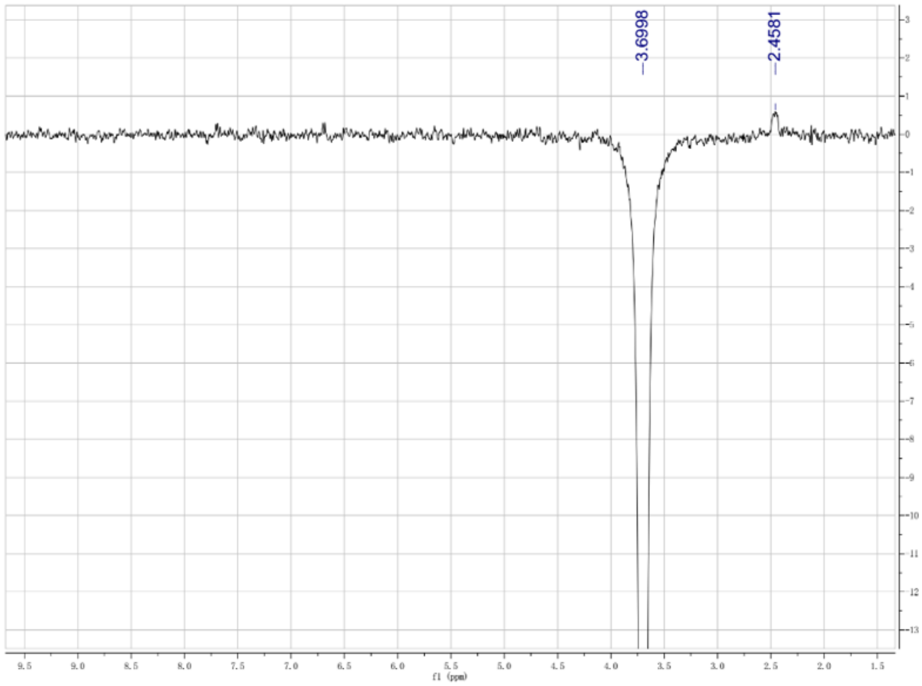

No.	Parameter	Value
1	Origin	Varian
2	Instrument	vnmrs
3	Solvent	cdcl3
4	Volume of deuterated solvent	500 µL
5	Internal standard	TMS
6	Temperature	25
7	Pulse Sequence	NOESY1D
8	Experiment	1D-NOESY
9	Probe	OneP
10	Number of Scans	408
11	Receiver Gain	30
12	Relaxation Delay	1
13	Pulse Width	10.3
14	Acquisition Time	1.8219
15	Spectrometer Frequency	499.79
16	Spectral Width	8992.8
17	Lowest Frequency	-998
18	Nucleus	1H
19	Acquired Size	16384
20	Spectral Size	65536

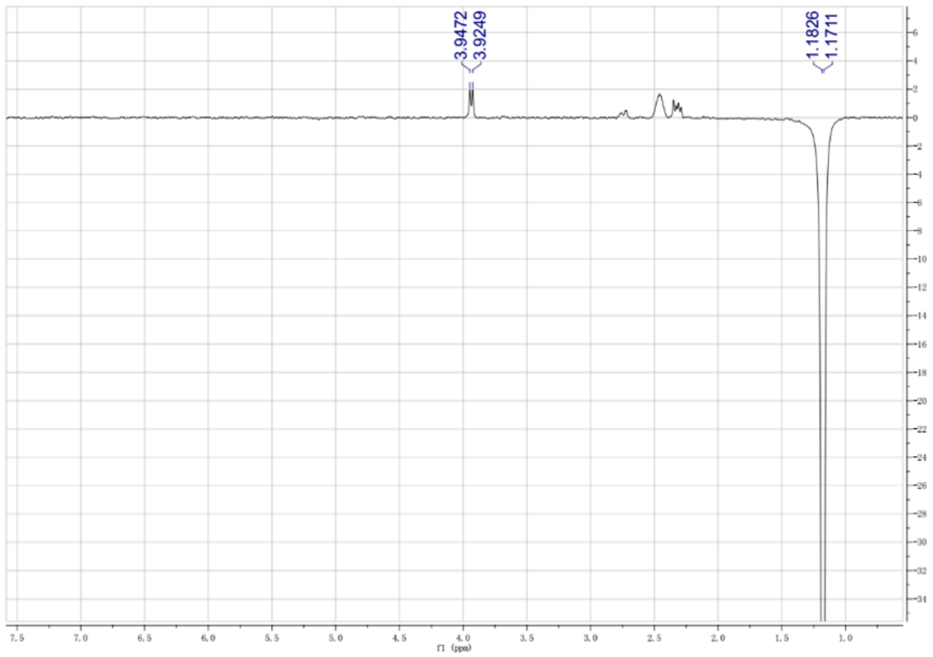

No.	Parameter	Value
1	Origin	Varian
2	Instrument	vnmrs
3	Solvent	cdcl3
4	Volume of deuterated solvent	500 µL
5	Internal standard	TMS
6	Temperature	25
7	Pulse Sequence	NOESY1D
8	Experiment	1D-NOESY
9	Probe	OneP
10	Number of Scans	88
11	Receiver Gain	30
12	Relaxation Delay	1
13	Pulse Width	10.3
14	Acquisition Time	1.8219
15	Spectrometer Frequency	499.79
16	Spectral Width	8992.8
17	Lowest Frequency	-998
18	Nucleus	1H
19	Acquired Size	16384
20	Spectral Size	65536

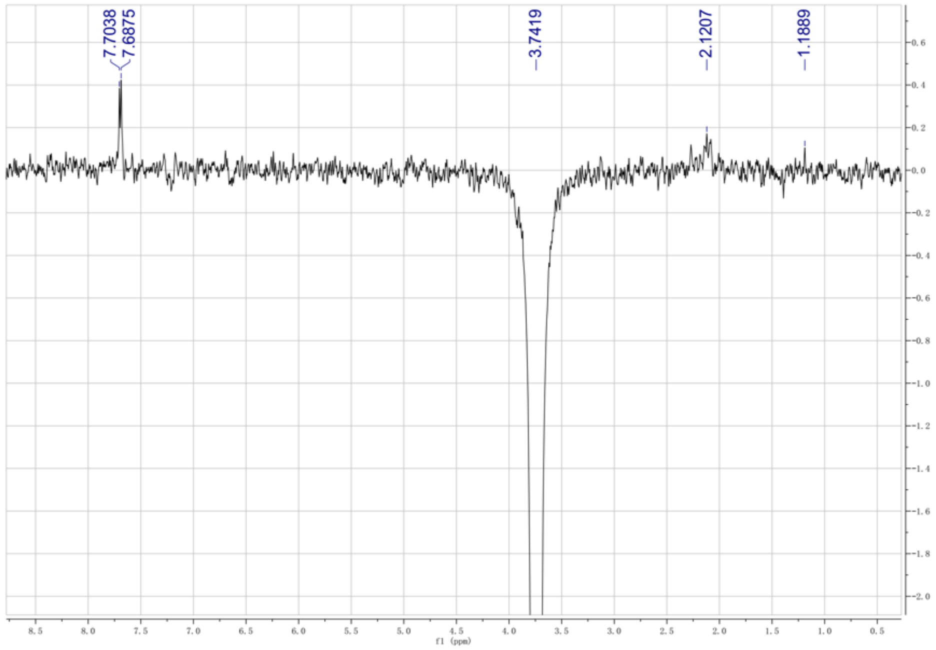

	Parameter	Value
1	Origin	Varian
2	Instrument	vnmrs
3	Solvent	cdcl3
4	Volume of deuterated solvent	500 µL
5	Internal standard	TMS
6	Temperature	25
7	Pulse Sequence	NOESY1D
8	Experiment	1D-NOESY
9	Probe	OneP
10	Number of Scans	216
11	Receiver Gain	30
12	Relaxation Delay	1
13	Pulse Width	10.3
14	Acquisition Time	1.8219
15	Spectrometer Frequency	499.79
16	Spectral Width	8992.8
17	Lowest Frequency	-998
18	Nucleus	1H
19	Acquired Size	16384
20	Spectral Size	65536

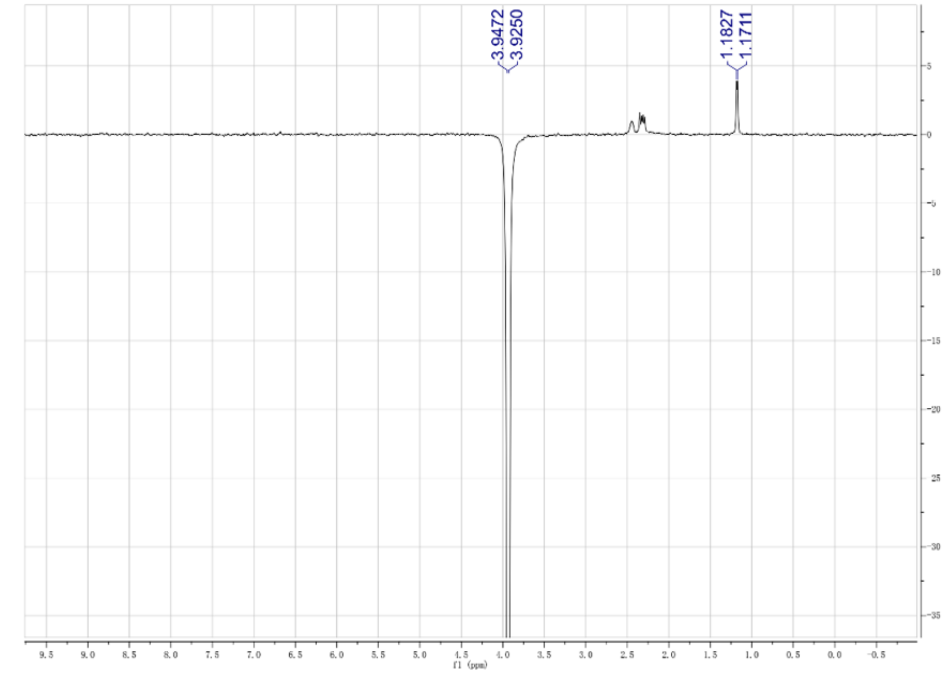

No.	Parameter	Value
1	Origin	Varian
2	Instrument	vnmrs
3	Solvent	cdcl3
4	Volume of deuterated solvent	500 μL
5	Internal standard	TMS
6	Temperature	25
7	Pulse Sequence	NOESY1D
8	Experiment	1D-NOESY
9	Probe	OneP
10	Number of Scans	120
11	Receiver Gain	30
12	Relaxation Delay	1
13	Pulse Width	10.3
14	Acquisition Time	1.8219
15	Spectrometer Frequency	499.79
16	Spectral Width	8992.8
17	Lowest Frequency	-998
18	Nucleus	1H
19	Acquired Size	16384
20	Spectral Size	65536

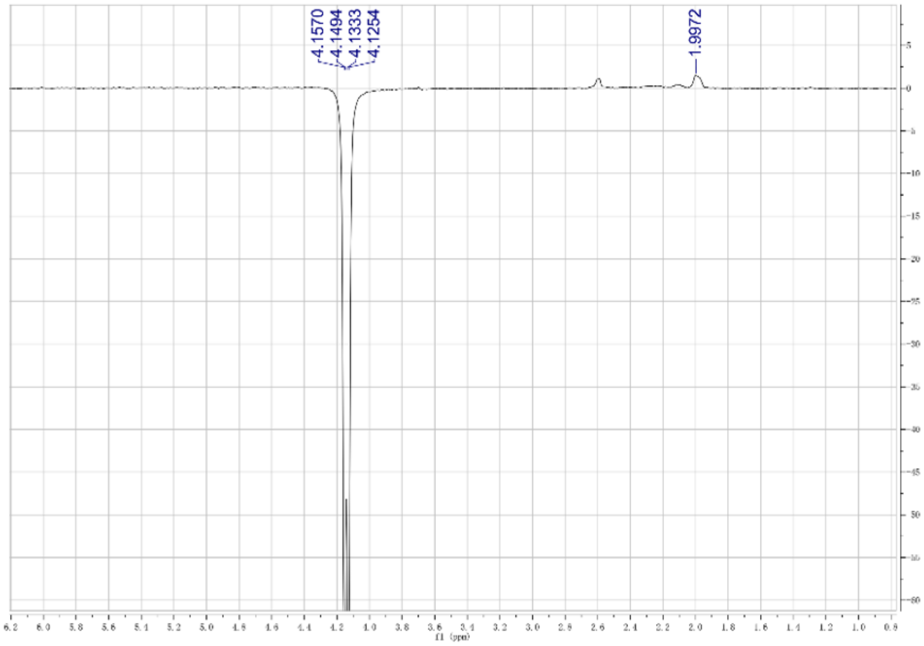

No.	Parameter	Value
1	Origin	Varian
2	Instrument	vnmrs
3	Solvent	cdcl3
4	Volume of deuterated solvent	500 μL
5	Internal standard	TMS
6	Temperature	25
7	Pulse Sequence	NOESY1D
8	Experiment	1D-NOESY
9	Probe	OneP
10	Number of Scans	64
11	Receiver Gain	30
12	Relaxation Delay	1
13	Pulse Width	10.3
14	Acquisition Time	1.8219
15	Spectrometer Frequency	499.79
16	Spectral Width	8992.8
17	Lowest Frequency	-998
18	Nucleus	1H
19	Acquired Size	16384
20	Spectral Size	65536

Figure S14. HRESIMS spectrum of versixanthone N (**2**)

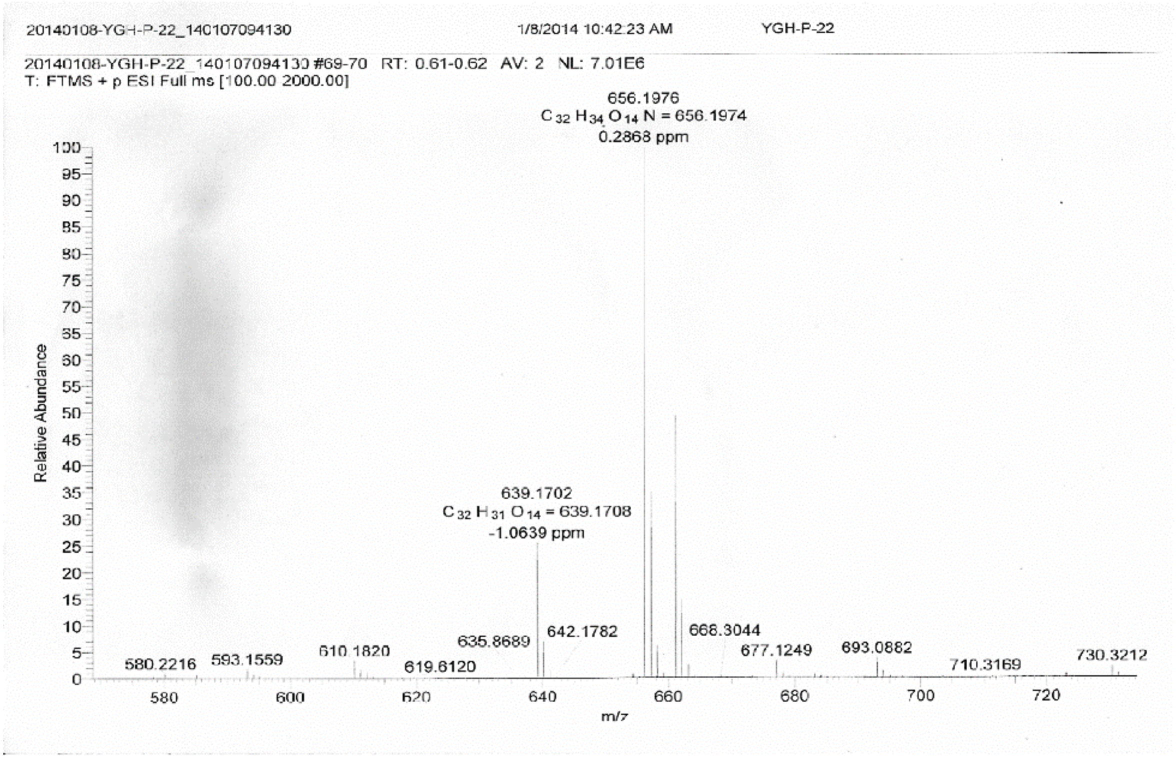

Figure S15. ^{1}H NMR spectrum (500 MHz) of versixanthone O (**3**) in $CDCl_3$

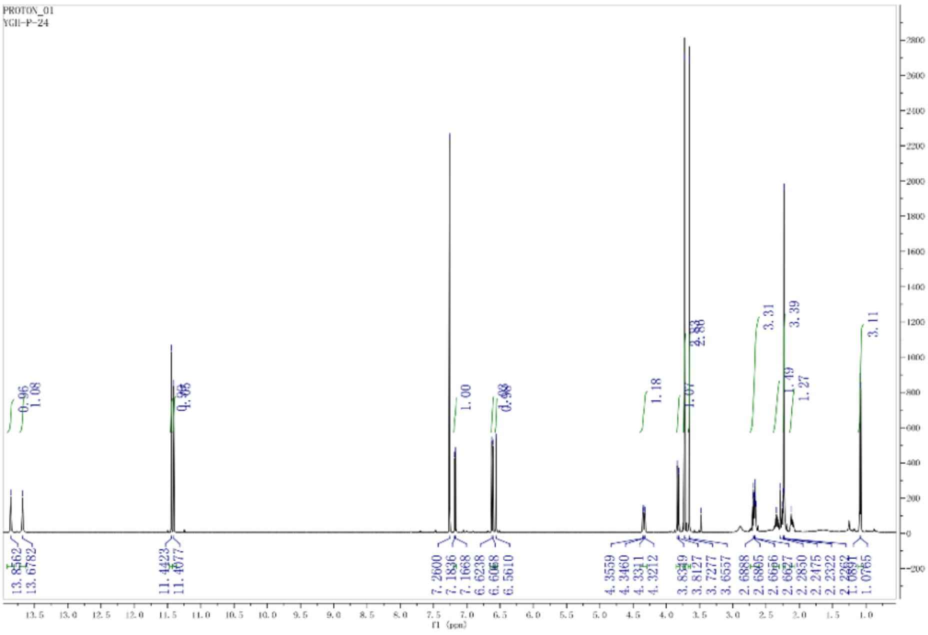

No.	Parameter	Value
1	Origin	Varian
2	Instrument	vnmrs
3	Solvent	cdcl3
4	Volume of deuterated solvent	500 µL
5	Internal standard	TMS
6	Temperature	25
7	Pulse Sequence	s2pul
8	Experiment	1D
9	Probe	OneP
10	Number of Scans	16
11	Receiver Gain	50
12	Relaxation Delay	1
13	Pulse Width	5.15
14	Acquisition Time	2.0447
15	Spectrometer Frequency	499.79
16	Spectral Width	8012.8
17	Lowest Frequency	-1007.7
18	Nucleus	1H
19	Acquired Size	16384
20	Spectral Size	65536

Figure S16. ^{13}C NMR spectrum (125 MHz) of versixanthone O (**3**) in $CDCl_3$

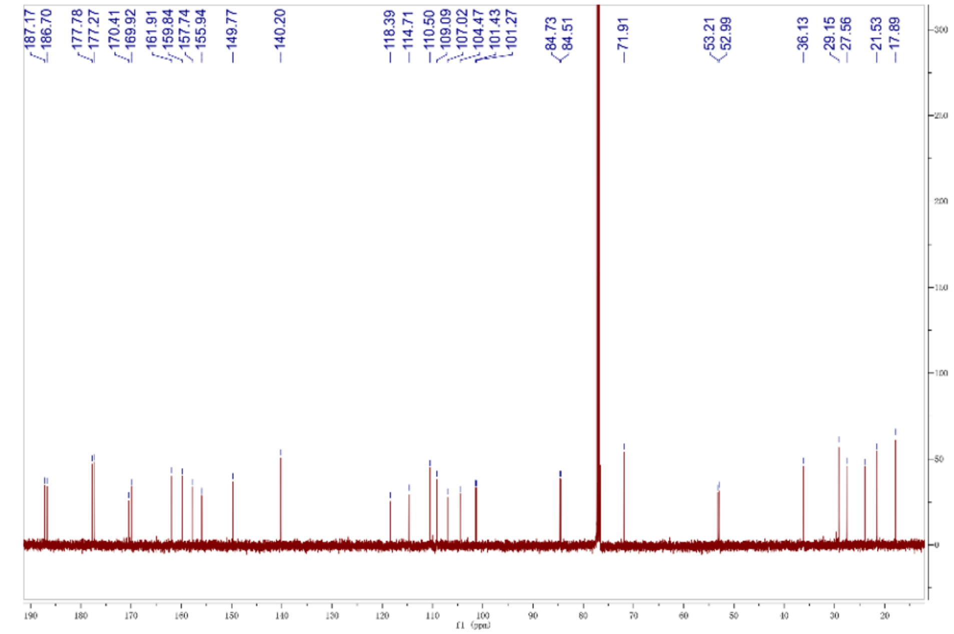

No.	Parameter	Value
1	Origin	Varian
2	Instrument	vnmrs
3	Solvent	cdcl3
4	Volume of deuterated solvent	500 µL
5	Internal standard	TMS
6	Temperature	25
7	Pulse Sequence	s2pul
8	Experiment	1D
9	Probe	OneP
10	Number of Scans	10000
11	Receiver Gain	30
12	Relaxation Delay	1
13	Pulse Width	4.95
14	Acquisition Time	1.0486
15	Spectrometer Frequency	125.68
16	Spectral Width	31250
17	Lowest Frequency	-1801.2
18	Nucleus	13C
19	Acquired Size	32768
20	Spectral Size	65536

Figure S17. HSQC spectrum of versixanthone O (**3**)

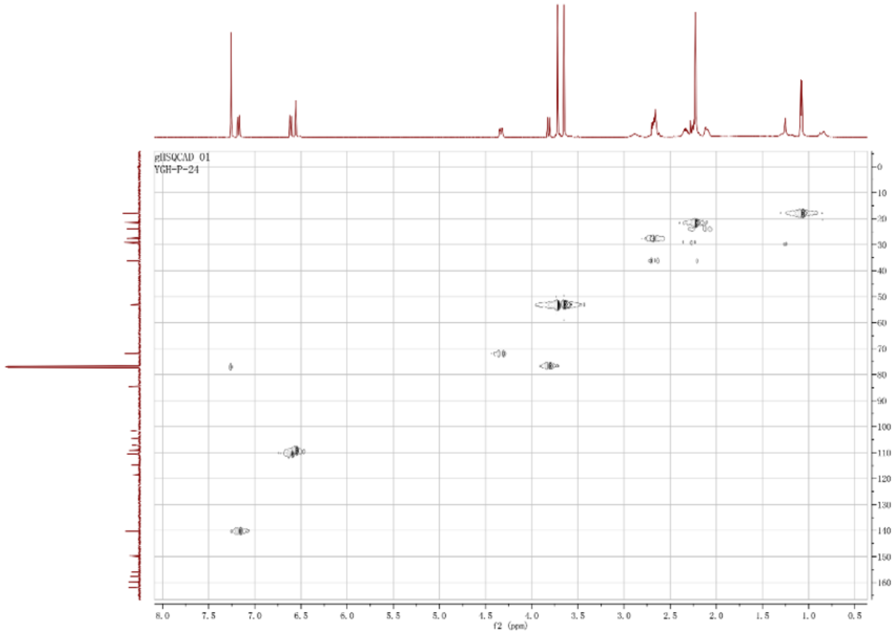

No.	Parameter	Value
1	Origin	Varian
2	Instrument	vnmrs
3	Solvent	cdcl3
4	Volume of deuterated solvent	500 µL
5	Internal standard	TMS
6	Temperature	25
7	Pulse Sequence	gHSQCAD
8	Experiment	HSQC-EDITED
9	Probe	OneP
10	Number of Scans	48
11	Receiver Gain	30
12	Relaxation Delay	1
13	Pulse Width	10.3
14	Acquisition Time	0.15
15	Spectrometer Frequency	(499.79, 125.68)
16	Spectral Width	(8012.8, 25133.5)
17	Lowest Frequency	(-1007.7, -1256.4)
18	Nucleus	(1H, 13C)
19	Acquired Size	(1202, 96)
20	Spectral Size	(2048, 1024)

Figure S18. ^{1}H-^{1}H COSY spectrum of versixanthone O (**3**)

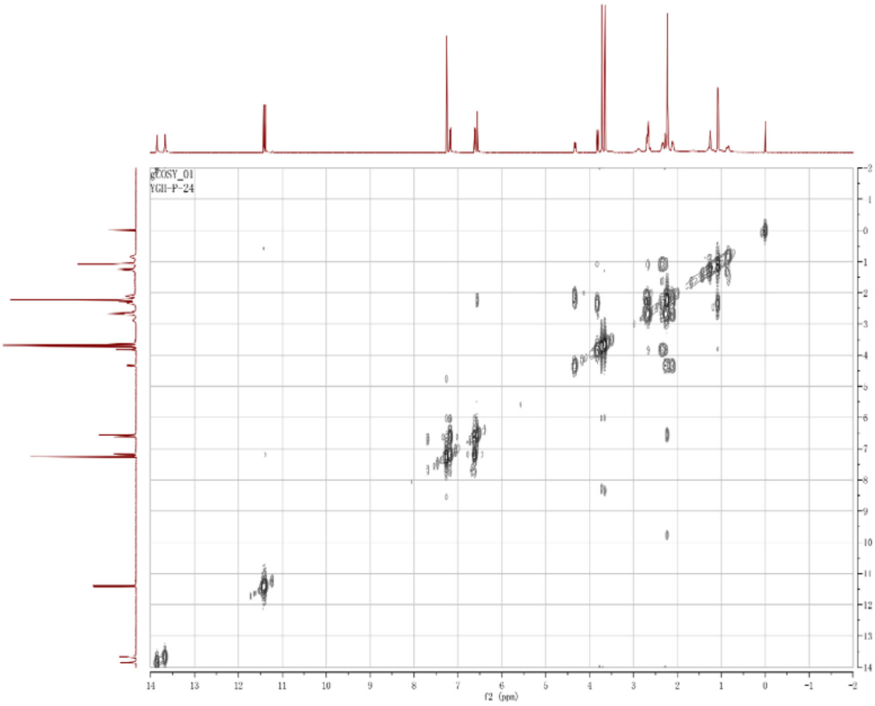

No.	Parameter	Value
1	Origin	Varian
2	Instrument	vnmrs
3	Solvent	cdcl3
4	Volume of deuterated solvent	500 µL
5	Internal standard	TMS
6	Temperature	25
7	Pulse Sequence	gCOSY
8	Experiment	COSY
9	Probe	OneP
10	Number of Scans	16
11	Receiver Gain	30
12	Relaxation Delay	1
13	Pulse Width	10.3
14	Acquisition Time	0.15
15	Spectrometer Frequency	(499.79, 499.79)
16	Spectral Width	(8012.8, 8012.8)
17	Lowest Frequency	(-1007.7, -1007.7)
18	Nucleus	(1H, 1H)
19	Acquired Size	(1202, 128)
20	Spectral Size	(2048, 2048)

Figure S19. HMBC spectrum of versixanthone O (**3**)

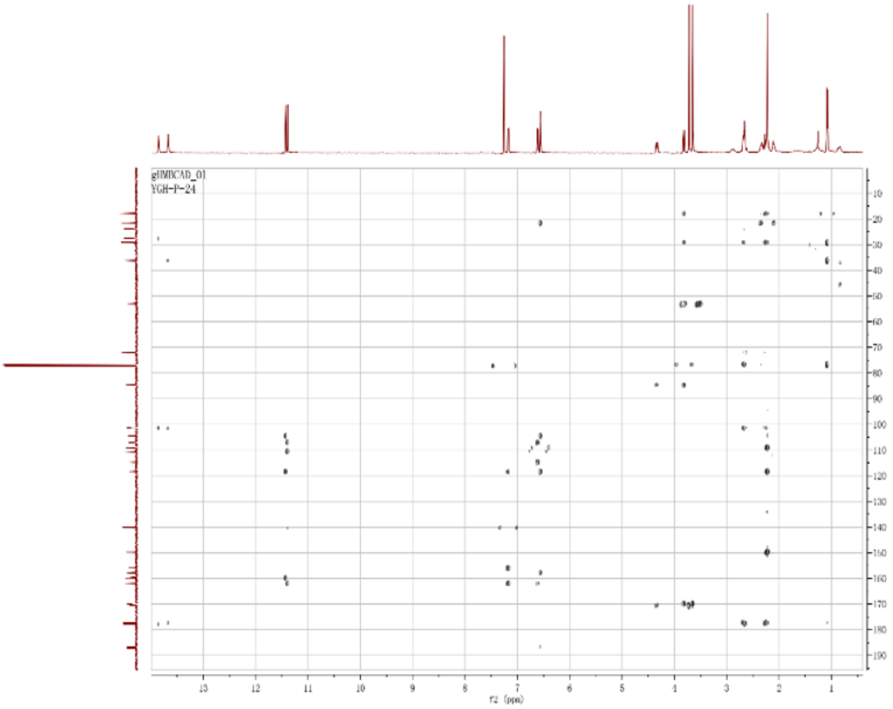

No.	Parameter	Value
1	Origin	Varian
2	Instrument	vnmrs
3	Solvent	cdcl3
4	Volume of deuterated solvent	500 µL
5	Internal standard	TMS
6	Temperature	25
7	Pulse Sequence	gHMBCAD
8	Experiment	HMBC
9	Probe	OneP
10	Number of Scans	36
11	Receiver Gain	30
12	Relaxation Delay	1
13	Pulse Width	10.3
14	Acquisition Time	0.15
15	Spectrometer Frequency	(499.79, 125.68)
16	Spectral Width	(8012.8, 30165.9)
17	Lowest Frequency	(-1007.7, -1887.6)
18	Nucleus	(1H, 13C)
19	Acquired Size	(1202, 200)
20	Spectral Size	(2048, 2048)

Figure S20. NOE spectrum of versixanthone O (**3**)

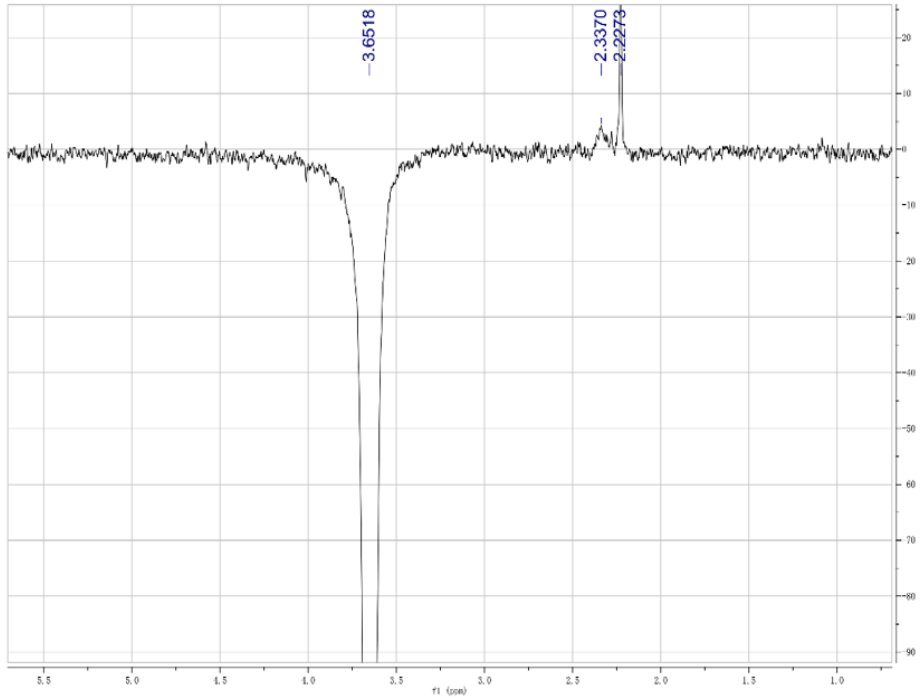

No.	Parameter	Value
1	Origin	Varian
2	Instrument	vnmrs
3	Solvent	cdcl3
4	Volume of deuterated solvent	500 µL
5	Internal standard	TMS
6	Temperature	25
7	Pulse Sequence	NOESY1D
8	Experiment	1D-NOESY
9	Probe	OneP
10	Number of Scans	656
11	Receiver Gain	48
12	Relaxation Delay	1
13	Pulse Width	10.3
14	Acquisition Time	2.0447
15	Spectrometer Frequency	499.79
16	Spectral Width	8012.8
17	Lowest Frequency	-1007.7
18	Nucleus	1H
19	Acquired Size	16384
20	Spectral Size	65536

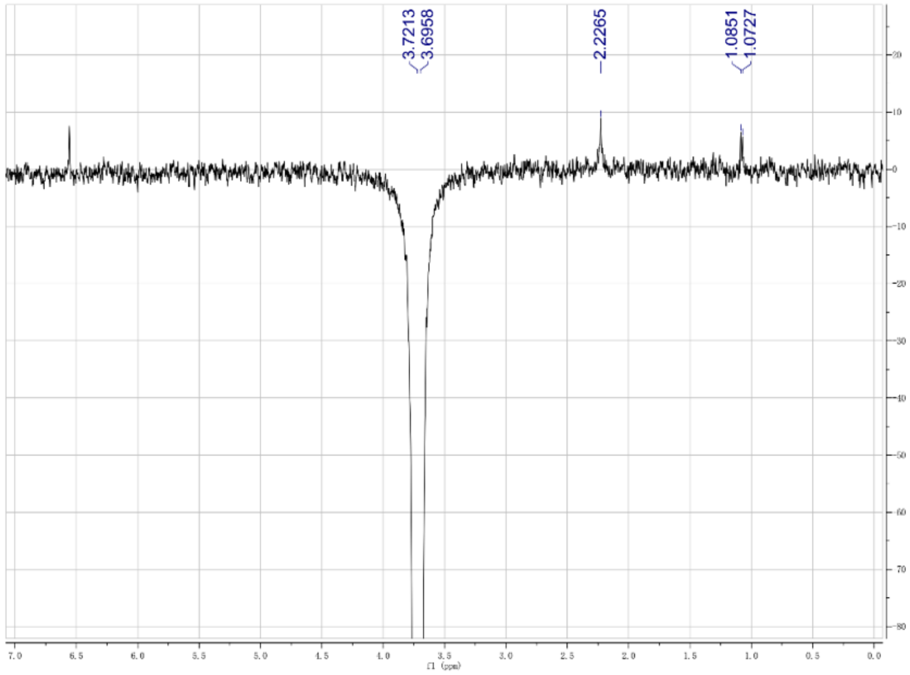

No.	Parameter	Value
1	Origin	Varian
2	Instrument	vnmrs
3	Solvent	cdcl3
4	Volume of deuterated solvent	500 µL
5	Internal standard	TMS
6	Temperature	25
7	Pulse Sequence	NOESY1D
8	Experiment	1D-NOESY
9	Probe	OneP
10	Number of Scans	800
11	Receiver Gain	48
12	Relaxation Delay	1
13	Pulse Width	10.3
14	Acquisition Time	2.0447
15	Spectrometer Frequency	499.79
16	Spectral Width	8012.8
17	Lowest Frequency	-1007.7
18	Nucleus	1H
19	Acquired Size	16384
20	Spectral Size	65536

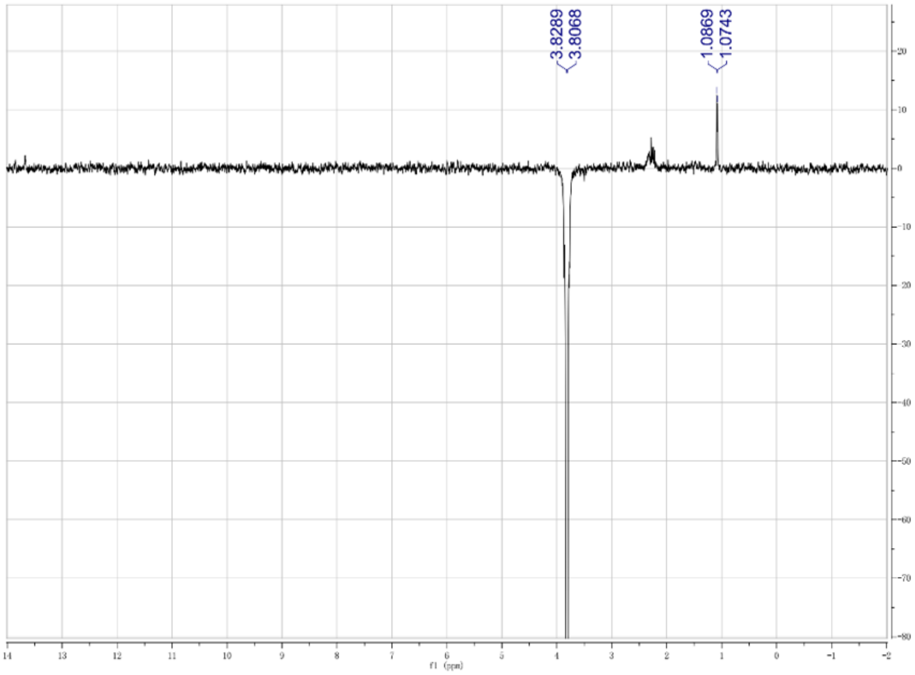

No.	Parameter	Value
1	Origin	Varian
2	Instrument	vnmrs
3	Solvent	cdcl3
4	Volume of deuterated solvent	500 µL
5	Internal standard	TMS
6	Temperature	25
7	Pulse Sequence	NOESY1D
8	Experiment	1D-NOESY
9	Probe	OneP
10	Number of Scans	136
11	Receiver Gain	50
12	Relaxation Delay	1
13	Pulse Width	10.3
14	Acquisition Time	2.0447
15	Spectrometer Frequency	499.79
16	Spectral Width	8012.8
17	Lowest Frequency	-1007.7
18	Nucleus	1H
19	Acquired Size	16384
20	Spectral Size	65536

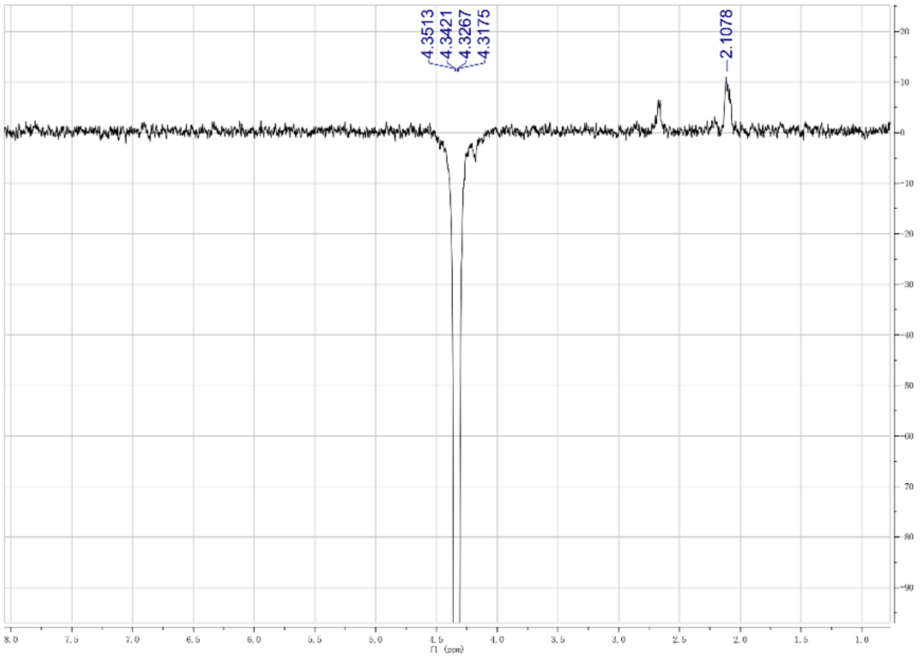

No.	Parameter	Value
1	Origin	Varian
2	Instrument	vnmrs
3	Solvent	cdcl3
4	Volume of deuterated solvent	500 µL
5	Internal standard	TMS
6	Temperature	25
7	Pulse Sequence	NOESY1D
8	Experiment	1D-NOESY
9	Probe	OneP
10	Number of Scans	224
11	Receiver Gain	50
12	Relaxation Delay	1
13	Pulse Width	10.3
14	Acquisition Time	2.0447
15	Spectrometer Frequency	499.79
16	Spectral Width	8012.8
17	Lowest Frequency	-1007.7
18	Nucleus	1H
19	Acquired Size	16384
20	Spectral Size	65536

Figure S21. HRESIMS spectrum of versixanthone O (**3**)

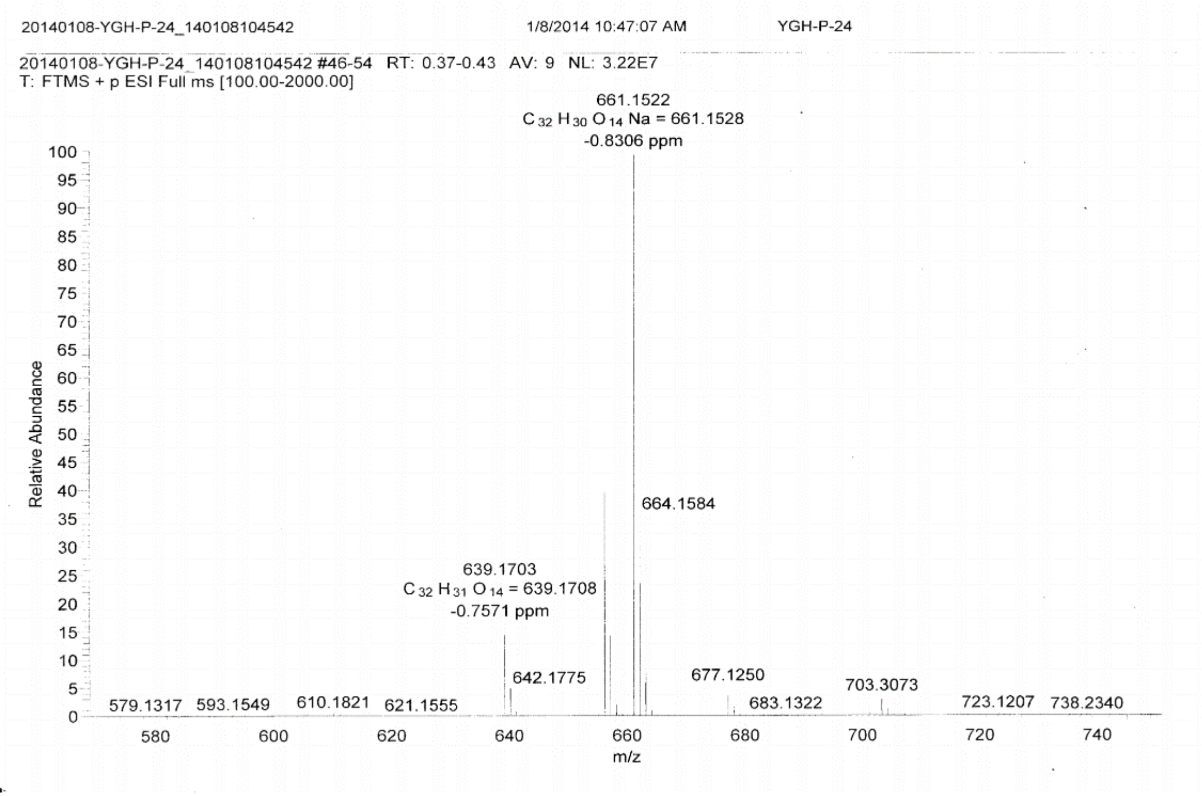

Supplement: Supplementary file 1 [file marinedrugs-16-00335-s001.pdf]
